# Mediterranean Diet Adherence, Fatty Acid Profiles, and Early Assisted Reproduction Outcomes: Findings from a 12-Week Dietary Intervention

**DOI:** 10.3390/medicina62030539

**Published:** 2026-03-13

**Authors:** Özge Cemali, Yasemin Akdevelioğlu, Aysel Berkkan, Onur Kenan Ulutaş, Recep Onur Karabacak, İsmail Güler, Seyhan Gümüşlü

**Affiliations:** 1 Department of Nutrition and Dietetics, Faculty of Health Science, Trakya University, 22020 Edirne, Türkiye; 2 Department of Nutrition and Dietetics, Faculty of Health Science, Gazi University, 06500 Ankara, Türkiye; 3 Department of Analytical Chemistry, Faculty of Pharmacy, Gazi University, 06330 Ankara, Türkiye; 4 Department of Pharmaceutical Toxicology, Faculty of Pharmacy, Gazi University, 06330 Ankara, Türkiye; 5 Department of Gynecology and Obstetrics, Faculty of Medicine, Surgical Medical Sciences, Gazi University, 06500 Ankara, Türkiye; 6 Faculty of Medicine, IVF Center, Gazi University, 06500 Ankara, Türkiye

**Keywords:** fatty acids, follicular fluid, implantation, infertility, mediterranean diet, ratio of ω6/ω3 fatty acid

## Abstract

*Background and Objectives*: Evidence on the impact of adherence to the Mediterranean diet alone—without supplementation—on serum and follicular fluid fatty acid profiles and assisted reproduction outcomes remains limited. This study evaluated the effects of a pre-treatment Mediterranean diet intervention on these parameters. *Materials and Methods:* In this prospective, non-randomized controlled trial, 32 women undergoing infertility treatment were allocated to a Mediterranean diet intervention group (*n* = 16) or a control group (*n* = 16). The intervention lasted 12 weeks, and adherence was assessed using validated dietary indices. Serum and follicular fluid fatty acid profiles were analyzed, and implantation and pregnancy outcomes were recorded. *Results:* The diet group showed increased ω-6 and ω-3 intake with decreased LA/ALA and ω-6/ω-3 ratios. In the control group, serum EPA + DHA levels declined, whereas in the diet group serum LA/ALA decreased. Follicular fluid in the intervention group had lower EPA + DHA and ω-6 ratios. Diet adherence correlated positively with MII oocytes (r = 0.797) and pronuclei (r = 0.741). No significant associations were found between follicular fluid fatty acids and IVF outcomes. A total of four implantation events were observed (intervention: *n* = 3; control: *n* = 1). Two of the implantations in the intervention group resulted in live births, while the remaining implantation events did not result in live birth. *Conclusions:* A Mediterranean diet-aligned dietary intervention may induce favorable changes in blood and follicular fluid parameters; however, the underlying metabolic mechanisms warrant further investigation. Three implantations were observed in the intervention group and one in the control group; given the low number of events, comparisons of live birth outcomes should be interpreted with caution. Overall, the findings regarding ART outcomes and clinical translation remain exploratory due to the limited sample size. Well-designed randomized controlled trials in which ART and clinical endpoints are defined as primary outcomes are needed.

## 1. Introduction

The Mediterranean diet (MD), originating from countries around the Mediterranean basin, is characterized by a high intake of plant-based foods such as fruits, vegetables, whole grains, legumes, nuts, and olive oil, together with moderate amounts of fish, poultry, eggs, and dairy products [1,2]. The Mediterranean diet is rich in antioxidants, fiber, vitamins, and healthy fats such as monounsaturated (MUFA) and polyunsaturated fatty acids (PUFA), which exert anti-inflammatory and antioxidative effects that are relevant to reproductive health [3,4]. Infertility, recognized by the World Health Organization as a disease, affects approximately 15% of couples of reproductive age worldwide, with increasing prevalence linked to delayed parenthood and lifestyle changes. Assisted reproductive technologies (ART), including in vitro fertilization (IVF) and intracytoplasmic sperm injection (ICSI), provide an important option for these couples, yet success rates remain limited, with live birth rates per cycle ranging from 30% to 40%. Given the invasiveness, cost, and psychological burden of ART, complementary and easily applicable lifestyle strategies, such as dietary interventions, are increasingly recognized as potential modulators of treatment success [5,6,7].

Growing evidence suggests that adherence to the MD may improve fertility outcomes. Epidemiological studies have shown positive associations between MD adherence and sperm quality, oocyte competence, and embryo development [8,9]. In particular, antioxidants, vitamins, and ω-3 fatty acids derived from the MD are thought to optimize the follicular environment, reduce oxidative stress, and support endometrial receptivity [10,11]. Recent systematic reviews reinforce this notion, reporting that couples adhering to the MD during ART cycles achieved higher numbers of embryos, improved implantation rates, and greater chances of clinical pregnancy and live birth, although some inconsistencies remain across studies [7]. Several studies in the literature have retrospectively evaluated adherence to the Mediterranean diet and reported its positive effects on the success of assisted reproductive treatment (ART) [1,2,3,4,12,13,14]. Additionally, there is evidence linking blood and follicular fluid fatty acid parameters with ART outcomes [15,16]. However, only a limited number of studies have explored the relationship between nutritional status, fatty acid profiles in blood and follicular fluid, and infertility treatment outcomes [14,17,18] and among them, only one study involved an actual dietary intervention [17].

To the best of our knowledge, no previous study has evaluated serum and follicular fluid fatty acid profiles following a diet-only intervention in the pre-ART period. Accordingly, the primary aim of this study was to investigate the association between adherence to the Mediterranean diet during the three-month preconception period and fatty acid profiles in serum and follicular fluid samples. The secondary aim was to assess the relationship between Mediterranean diet adherence, serum and follicular fluid fatty acid profiles, and ART outcomes. Implantation success and live birth outcomes were also recorded and are presented as exploratory clinical endpoints.

## 2. Materials and Methods

### 2.1. Ethical Approval and Research Funding

The studies involving humans were approved by the Gazi University Clinical Research Ethics Committee, Faculty of Medicine, Ankara, Türkiye (Approval No: 87, Date: 25 October 2019). The study was conducted in accordance with local legislation, institutional requirements, and the ethical standards of the national research committee, as well as with the 1964 Helsinki Declaration and its later amendments.

Written informed consent was obtained from all participants prior to inclusion in the study. Participant recruitment was conducted between February 2021 and February 2022.

The clinical trial was registered retrospectively under the number NCT06559904 on 16 August 2024. The study design, primary and secondary outcome measures, and statistical analysis plan were defined a priori before the initiation of data collection, and no post hoc modifications to the outcomes were made.

This research was supported by the Gazi University Scientific Research Projects Unit as part of an Independent Research Project (Project Code: 47/2020-06).

### 2.2. Study Desing and Setting

This study was designed as a single-center, non-randomized, controlled, prospective intervention trial. It was conducted between February 2021 and February 2022 with voluntary female participants who applied to the In Vitro Fertilization/Intracytoplasmic Sperm Injection [IVF-ICSI] Outpatient Clinic of the Department of Obstetrics and Gynecology at Gazi University. Biological samples were stored in the Medical Biochemistry Laboratory of Gazi University until the time of analysis and were analyzed at the Faculty of Pharmacy, Gazi University. All biological samples were stored in a single laboratory, and laboratory conditions were maintained consistently throughout the storage period. Samples were frozen and preserved under identical temperature and storage conditions in accordance with a standardized protocol, with no protocol modifications made prior to analysis. During the analytical phase, all samples were transported under uniform conditions to the gas chromatography (GC) laboratory located approximately five minutes from the storage facility. Throughout the study period, no changes were made to the clinical protocols related to IVF or insemination procedures; embryology and laboratory processes were conducted within the same center using consistent technical infrastructure. This approach ensured methodological consistency across storage, transport, analytical procedures, and clinical applications.

### 2.3. Participants

**Figure 1:**
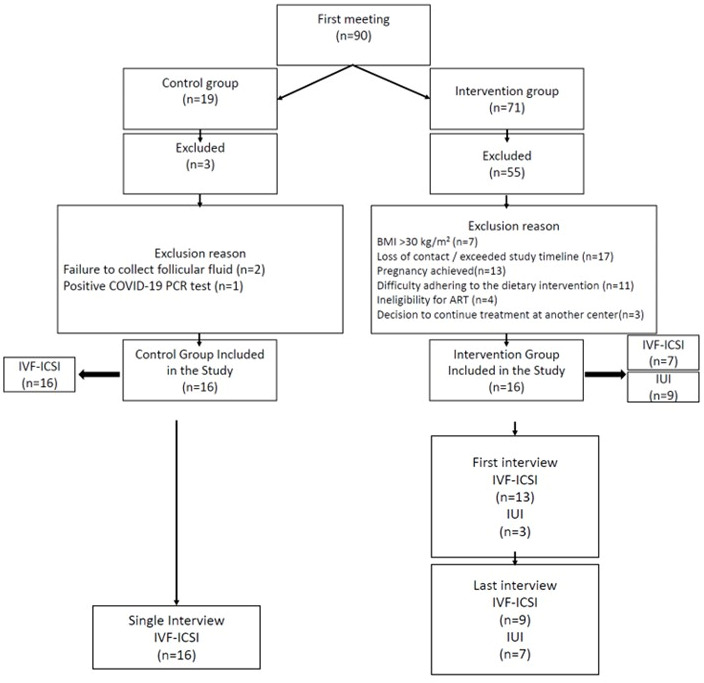
Participant flow diagram according to the TREND statement.

Women aged 19–40 years, with BMI < 30 kg/m^2^, without chronic disease, and not using tobacco or alcohol, who consecutively applied to the IVF-ICSI Outpatient Clinic and met the eligibility criteria, were invited to participate. Both the intervention and control groups were selected from the same pool of applicants within the study period; no external group was used. Exclusion criteria (e.g., BMI > 30 kg/m^2^, chronic illness, positive COVID-19 PCR, pregnancy during the study period, or failure to provide follicular fluid samples) are detailed in Figure 1 and summarized here for clarity. Figure 1 presents the flow of participants through enrollment, assignment, follow-up, and analysis in accordance with the TREND reporting guidelines. Infertility diagnoses included both female and male factors; women with unexplained infertility were also included.

The minimum required sample size was determined based on similar studies [18,19] and calculated through an a priori power analysis (α = 0.05, power = 0.95), targeting the primary objective of the study. Accordingly, at least 15 participants per group (total n = 30) were required. The study was completed with 32 women. The sample size calculation was specifically based on the primary endpoint; namely, the evaluation of the association between Mediterranean diet adherence during the preconception period and serum and follicular fluid fatty acid profiles.

Follicular fluid samples were obtained from a smaller subset (*n* = 7 in the intervention group) due to feasibility and consent limitations. Therefore, findings related to secondary objectives, particularly ART outcomes and clinical endpoints, should be interpreted as exploratory in nature.

### 2.4. Intervention Protocol

The intervention group received a Mediterranean diet-based nutrition program for 12 weeks, consisting of five consultations. The control group received two standard consultations. Due to the inherent nature of IVF treatment, which is typically completed within a 12–14-day cycle, enrolling the control group at the beginning, middle, or end of the study would have corresponded only to a 12–14-day segment of the intervention group’s three-month dietary period. Therefore, participants were recruited sequentially, with the intervention group enrolled first, followed by the control group.

**Figure 2:**
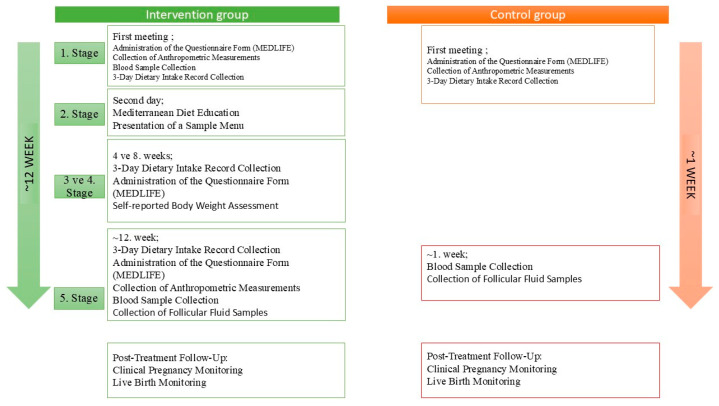
Overview of Study Procedures.

The mean duration between the first and final consultations in the diet (intervention) group was 15.03 ± 1.59 weeks, whereas the interval between consultations in the control group was 1.41 ± 0.53 weeks. This sequential recruitment strategy was implemented due to feasibility considerations and allowed completion of the study within the planned timeframe; however, the absence of randomization constitutes a methodological limitation. A detailed overview of the study protocol is presented in Figure 2.

### 2.5. Data Collection Methods and Instruments

Baseline demographic, infertility-related, and clinical data were obtained via structured questionnaires and medical records. Dietary intake was assessed using repeated 3-day food records, analyzed with the Nutrition Information System, BeBis, version9 (Istanbul, Türkiye). Adherence to the Mediterranean diet was evaluated using the MEDLIFE index, complemented by the Mediterranean Diet Quality Index (MDQI). Physical activity was measured with the validated IPAQ-SF [20]. Anthropometric measurements (weight, height, waist/hip circumference, body composition, and BMR) were recorded at baseline and follow-up visits. Detailed instruments and equations (e.g., BMR calculation, IPAQ classification, scoring methods [21,22,23]) are provided in Tables S1–S3.

### 2.6. Assessment of Dietary Intake

Participants’ dietary intake was assessed using 3-day food consumption records, covering two weekdays and one weekend day. These records were collected by the researcher once in the control group and four times in the intervention group. In the intervention group, the first and final records were obtained through face-to-face interviews, while dietary intake at weeks 4 and 8 was assessed via telephone interviews. During the initial interview, participants were trained to estimate portion sizes accurately using the “Photograph Catalog of Foods and Meals,” which was adapted to common household measurements (e.g., cups, plates, bowls, spoons). Daily energy and nutrient intake values were calculated using the full version of the Nutrition Information System BeBis, version9 (Istanbul, Türkiye).

### 2.7. Mediterranean Diet Intervention

In the intervention group, participants were provided with a Mediterranean diet-based nutrition program for a duration of 12 weeks. The dietary plans were individually tailored according to each participant’s energy requirements, with no caloric restriction imposed.

### 2.8. Mediterranean Diet: Food and Nutrient Composition

Participants received sample menus with energy levels ranging between 1600 and 1900 kcal/day (Table S2). The determination of daily and weekly portion sizes for food groups was guided by the MEDLIFE index. In addition to the recommendations listed in Table 1, the sample menus also included daily and weekly target portion amounts (Table S2) consistent with the Mediterranean Diet, as well as lifestyle recommendations aligned with the Mediterranean Lifestyle (Table S3), which participants were encouraged to follow throughout the intervention.

Assessment of Diet Adherence: During the four follow-up visits conducted in the intervention group, participants were asked about the frequency with which they consumed food items emphasized in the MEDLIFE index on a weekly basis. Their adherence to the Mediterranean diet was evaluated using the MEDLIFE scoring system.

The MEDLIFE index was developed in accordance with the principles of the Mediterranean Diet Pyramid proposed by the Mediterranean Diet Foundation of Spain in 2011. The index comprises 28 items divided into three blocks. The first block includes 15 items related to the frequency of food consumption. The second block consists of 7 items evaluating Mediterranean dietary habits. The third block contains 6 items addressing physical activity, rest, social habits, and leisure activities. Each item is scored as either 0 or 1, resulting in a total score ranging from 0 (poorest adherence) to 28 (highest adherence) [24]. The scale does not include a predefined cut-off point. In line with prior studies showing variation in adherence definitions [6,7], we pragmatically defined adherence in this study as achieving ≥50% of the maximum score on the MEDLIFE index. This threshold was selected to differentiate participants with moderate-to-high adherence in absence of an established gold-standard cut-off. In this study, women in the intervention group were considered adherent to the dietary program if their total score exceeded 50% at each follow-up visit.

In addition, during the study period, participants were asked to provide 3-day dietary records—including two weekdays and one weekend day—at baseline and at the 4th, 8th, and 12th weeks. At the end of the study, these dietary records were entered into the system. ystem. The following parameters were calculated using the program: total daily energy intake (kcal/day), macronutrients [carbohydrates (% kcal/day and g/day), protein (% kcal/day and g/day), fat (% kcal/day and g/day)], saturated fatty acids (SFA; % and g/day), monounsaturated fatty acids (MUFA; % and g/day), polyunsaturated fatty acids (PUFA; % and g/day), total ω-6 fatty acids, total ω-3 fatty acids, ω-6/ω-3 fatty acid ratio, linoleic acid/alpha-linolenic acid (LA/ALA) ratio, and total %EPA + DHA.

Based on the foods recorded in the dietary intake logs, the daily consumption amounts of food groups assessed in the MEDLIFE index were calculated. These included dairy products (milk, yogurt, cheese; g/day), red and poultry meats (g/day), fish (g/day), eggs (g/day), legumes (g/day), bread and cereals (g/day), olives and nuts (g/day), fruits (g/day), vegetables (g/day), and olive oil (g/day).

Finally, in addition to assessing adherence to the Mediterranean diet program, the daily consumption amounts obtained from dietary intake records were also evaluated using the Mediterranean Diet Quality Index (MDQI), developed by Gerber to assess adherence to the Mediterranean dietary pattern [24]. According to the MDQI, scores are interpreted as follows: 0–3 points = good adherence, 4–7 = moderate-good adherence, 8–11 = moderate-poor adherence, and 12–14 = poor adherence.

### 2.9. ART Protocols and Outcomes

Participants underwent IUI or IVF/ICSI depending on clinical indication. ART protocols and dosages were individualized, with details provided in Table S4 [25,26,27,28]. Clinical outcomes included number of retrieved oocytes, MII oocytes, fertilization and maturation rates, embryo transfer, implantation (β-hCG > 6 mIU/mL), clinical pregnancy (gestational sac on ultrasound), and live birth [28,29]. Detailed definitions of ART outcomes and clinical endpoints are also clearly described in the Supplementary File. This approach was adopted to enhance transparency and minimize potential interpretative bias.

### 2.10. Biological Sampling and Fatty Acid Analysis

Fasting blood and follicular fluid samples were collected, centrifuged, and stored at −80 °C until analysis. Storage conditions were strictly standardized and maintained consistently throughout the study period, with all samples preserved under identical temperature-controlled conditions and without protocol deviations. Fatty acid profiles were determined using gas chromatography with flame ionization detection (GC-FID). Details of sample preparation and calculated fatty acid parameters are available in Tables S5 and S6.

### 2.11. Statistical Analysis and Data Evaluation

Categorical variables were presented as frequency (*n*) and percentage (%), while continuous variables were described using mean ($\bar{x}$), standard deviation (SD), median, and interquartile range (IQR). Differences between categorical variables were analyzed using the Chi-square test. The distribution of continuous data was assessed using histograms, coefficient of variation, skewness, kurtosis, and the Kolmogorov–Smirnov test. For between-group comparisons, the Mann–Whitney U test was used for non-normally distributed variables, and the Wilcoxon signed-rank test was applied for within-group comparisons. Relationships between continuous variables were analyzed using Spearman’s correlation coefficient. A *p*-value of < 0.05 was considered statistically significant. All analyses were performed using SPSS version 22.0 (IBM Inc., Chicago, IL, USA). Effect estimates (odds ratios) with 95% confidence intervals were calculated for key clinical endpoints. Given the low number of events, these analyses were considered exploratory and potentially imprecise. For outcomes with sparse data or zero cell counts, exact methods were applied to estimate odds ratios and corresponding confidence intervals.

## 3. Results

### 3.1. BaselineCharacteristics of the Participants

In the study, the mean age of participants was 27.6 ± 4.6 years in the intervention group (*n* = 16) and 29.8 ± 4.81 years in the control group (*n* = 16), with no statistically significant difference between groups (*p* > 0.05) (Table 2). Information on infertility characteristics, physical activity levels, and anthropometric measurements of individuals in both groups is presented in Table 2. No significant differences were found between the groups in terms of infertility-related variables, physical activity levels, or anthropometric parameters (Table 2). In both groups, 87.5% of women were diagnosed with primary infertility. A preliminary diagnosis of idiopathic infertility was present in approximately 62.5% of women in the intervention group and 75% in the control group (*p* > 0.05). Among female-related infertility causes, tubal factor, diminished ovarian reserve, oligoovulation, and hyperprolactinemia were noted, while male-related factors included teratozoospermia, azoospermia, oligozoospermia, oligoasthenoteratozoospermia, oligoteratozoospermia, and asthenozoospermia (*p* > 0.05).

Hormonal, biochemical, and hematological blood test results were obtained once at baseline for both the intervention and control groups and were within reference ranges; thus, they were not presented in the table.

### 3.2. Assessment of Participants’ Adherence to the Mediterranean Diet and Dietary Intake

The mean duration between the first and final consultations in the diet (intervention) group was 15.03 ± 1.59 weeks, while the interval between consultations in the control group was 1.41 ± 0.53 weeks. Data on participants’ adherence to the Mediterranean diet are presented in Table 3. Adherence was assessed using the MEDLIFE index and the Mediterranean Diet Quality Index (MDQI). A statistically significant difference was observed in MEDLIFE scores across the four follow-up visits within the intervention group (*p* < 0.05). Post hoc analyses revealed that the significant change occurred between the first (13.7 ± 3.86) and second (16.8 ± 1.73) visits (*p* = 0.012). When pre- and post-intervention scores were compared (13.7 ± 3.86 vs. 15.9 ± 2.47), MEDLIFE scores increased after the intervention (*p* < 0.05). At the final evaluation, MEDLIFE scores were significantly higher in the intervention group (15.9 ± 2.47) compared to the control group (11.0 ± 3.68) (*p* < 0.05), while the reverse-scored MDQI values were lower (5.1 ± 1.44 vs. 6.3 ± 1.74, respectively; *p* < 0.05). Three-day dietary records were collected four times in the intervention group (at baseline and every four weeks) and once in the control group. Macronutrient intake data derived from these records are summarized in Table 3. No statistically significant differences were observed between timepoints, except for the percentage of monounsaturated fatty acid (MUFA) intake, which was higher in the intervention group at the end of the study compared to the control group (*p* < 0.05). Food group-based intake amounts reflecting the Mediterranean dietary pattern are provided in Table S7. When final intake values were compared between the control and intervention groups, daily olive oil (9.5 ± 8.51 mL vs. 20.8 ± 9.35 mL) and legume (8.8 ± 8.28 g vs. 26.2 ± 20.90 g) consumption were found to be significantly higher in the intervention group (*p* < 0.05) (Table S7).

### 3.3. Dietary Record-Based Fatty Acid Estimates and Serum and Follicular Fluid Fatty Acid Levels Determined by GC Analysis

Fatty acid levels estimated from the dietary records of women in the control and intervention groups are presented in Table 4. No significant differences were observed in dietary fatty acid intake between the control group and the intervention group at baseline (*p* > 0.05). Additionally, comparisons between pre- and post-intervention values within the intervention group, as well as between the intervention group (post-intervention) and the control group, revealed no statistically significant differences in dietary fatty acid intake (*p* > 0.05).

**Figure 3:**
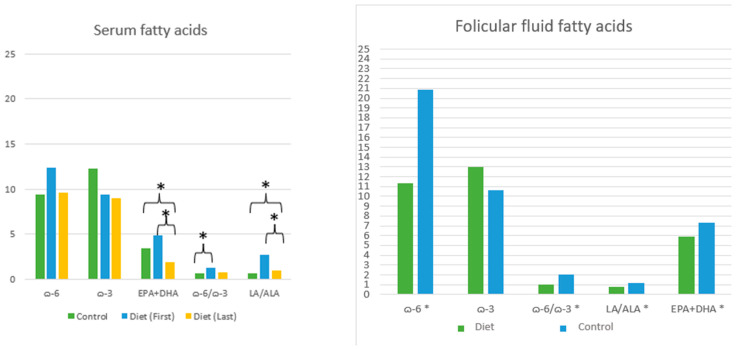
Serum and Follicular Fluid Fatty Acid Levels and Ratios; indicates statistically significant differences between groups.

Serum fatty acid analysis results are presented in Table 4 and Figure 3. At baseline, the intervention group had significantly higher LA/ALA and ω-6/ω-3 fatty acid ratios, and lower total ω-3 fatty acids and EPA + DHA levels compared to the control group (*p* < 0.05). At the end of the study, EPA + DHA levels remained lower in the intervention group compared to the control group (*p* < 0.05). When comparing pre- and post-intervention values within the intervention group, both EPA + DHA levels and the LA/ALA ratio were reduced by the end of the intervention (*p* < 0.05).

Follicular fluid fatty acid levels of participants are also shown in Table 4 and Figure 3. Compared to the control group, the intervention group showed lower levels of EPA + DHA, total ω-6 fatty acids, LA/ALA ratio, and ω-6/ω-3 fatty acid ratio in follicular fluid, with all differences reaching statistical significance (*p* < 0.05).

### 3.4. Association of Dietary, Serum, and Follicular Fluid Fatty Acid Levels with Adherence to the Mediterranean Diet Intervention

The correlations between serum fatty acid levels and MEDLIFE and MDQI scores are presented in Table 5. At the end of the intervention, a positive correlation was observed between MEDLIFE scores and total serum ω-6 fatty acid levels (*p* < 0.05). No significant associations were found between serum fatty acid levels and MDQI scores within the intervention group. Additionally, there were no significant correlations between follicular fluid fatty acid levels and either MEDLIFE or MDQI scores (*p* > 0.05).

### 3.5. Relationship Between Mediterranean Diet Adherence and IVF Outcomes

At baseline, 13 women in the intervention group were undergoing IUI and 3 were undergoing IVF treatment. By the end of the study, a significant difference was observed in the distribution of treatment protocols between the intervention and control groups. In the intervention group, 9 women had received IUI and 7 had received IVF treatment, whereas all women in the control group (*n* = 16) underwent IVF (*p* < 0.05).

IVF parameters and outcomes for both the intervention and control groups are presented in Table 6. Although no statistically significant differences were observed between groups, the total number of oocytes, number of metaphase II (MII) oocytes, and number of pronuclei were higher in the control group, while the maturation and fertilization rates were higher in the intervention group (*p* > 0.05). These findings correspond to secondary endpoints of the study and were not defined as primary clinical outcomes; as the sample size was not powered to detect differences in these parameters, they should be interpreted cautiously and considered exploratory in nature.

For clinical pregnancy, 3/16 events (18.8%) occurred in the intervention group compared to 1/16 (6.3%) in the control group (OR: 3.46; 95% CI: 0.32–37.04). For live birth, 2/16 events (12.5%) were observed in the intervention group, whereas no live births occurred in the control group (0/16). Using a continuity correction due to the zero cell count, the estimated odds ratio was 5.69 (95% CI: 0.25–129.4). Given the very low number of events, these estimates are exploratory and should be interpreted with caution. In the intervention group, two pregnancies (one singleton and one twin) resulted in live births.

No significant correlations were found between MEDLIFE scores and IVF parameters in either the intervention or control group (Table 7). However, in the intervention group, MDQI scores were positively correlated with the number of MII oocytes and the number of pronuclei (*p* < 0.05). No significant associations were observed between dietary fatty acid ratios and IVF parameters (*p* > 0.05). In the control group, serum levels of ω-3 polyunsaturated fatty acids were positively correlated with the number of MII oocytes and pronuclei (*p* < 0.05), while serum EPA and DHA levels showed a negative correlation with total oocytes, MII oocytes, and pronuclei (*p* < 0.05). In the intervention group, serum LA/ALA ratio was positively associated with the number of pronuclei and negatively associated with the maturation rate (*p* < 0.05).

As shown in Table 7, follicular fluid fatty acid correlations revealed that in the control group, increased levels of ω-3 fatty acids were associated with a decrease in total and MII oocytes (*p* < 0.05). Additionally, higher DHA levels in follicular fluid were associated with lower counts of total oocytes, MII oocytes, and pronuclei (*p* < 0.05).

### 3.6. Correlation of Dietary Intake with Serum and Follicular Fluid Fatty Acid Profiles

In participants with available follicular fluid samples (*n* = 23), associations among serum fatty acid levels, follicular fluid fatty acid levels, and dietary fatty acid intake were examined (Table 8). A moderate positive correlation was observed between serum EPA levels and dietary EPA intake (*p* < 0.05). Additionally, the follicular fluid LA/ALA ratio showed a moderate positive correlation with both dietary LA/ALA and ω-6/ω-3 fatty acid ratios (*p* < 0.05). Dietary EPA intake was also moderately and positively correlated with follicular fluid EPA levels (*p* < 0.05).

A significant negative association was observed between total serum and follicular fluid ω-6 fatty acid levels (*p* < 0.05). In contrast, serum and follicular fluid ω-3 fatty acid levels demonstrated a positive association (*p* < 0.05). Total serum EPA + DHA levels were positively associated with total follicular fluid ω-3 fatty acids, as well as with individual EPA and DHA levels (*p* < 0.05). However, total serum EPA + DHA levels were negatively associated with follicular fluid ω-6 fatty acid levels (*p* < 0.05).

Positive associations were also identified between serum and follicular fluid EPA levels and between serum and follicular fluid DHA levels. Serum DHA was positively associated with total follicular fluid EPA + DHA levels (*p* < 0.05). Additionally, serum EPA and DHA levels showed positive associations with follicular fluid ω-6 fatty acid levels (*p* < 0.05).

These findings suggest a potential biological relationship between serum and follicular fluid fatty acid profiles; however, causal inferences cannot be drawn.

## 4. Discussion

This study examined associations among dietary energy and macronutrient intake, MD adherence, serum and follicular fluid fatty acid profiles, IVF parameters, and reproductive outcomes—including implantation, clinical pregnancy, and live birth—in 32 volunteers.

Age, a major determinant of fertility [3], was similar between groups, minimizing age-related bias [19,20,21]. Fertility declines with advancing age, particularly in women, and markedly after 40 years. Therefore, only women aged ≤40 years were included, and comparable age distributions were ensured between groups, reducing potential age-related confounding. However, even within the ≤40 age range, subtle differences in ovarian aging dynamics may persist; thus, age-related residual confounding cannot be completely excluded.

No significant differences were found in socioeconomic or educational status (university graduates: 75.0% vs. 43.8%), hormone/biochemical/hemogram values, marriage and infertility duration, or infertility type (87.5% primary/idiopathic), supporting group comparability [13,30,31,32]. The similarity in baseline clinical, biochemical, and sociodemographic characteristics may have reduced the likelihood that major baseline confounding factors substantially influenced the outcomes; however, given the limited sample size and non-randomized design, residual and unmeasured confounding cannot be ruled out. Furthermore, the predominance of primary/idiopathic infertility in both groups may have limited etiological heterogeneity, thereby strengthening internal consistency. Nevertheless, this should not be interpreted as evidence that observed differences were exclusively attributable to dietary exposure.

Baseline physical activity was largely inactive in both groups (62.5% vs. 68.8%) [3,33], while BMI values were within normal ranges [5,15,31,32,34,35] and waist-to-hip ratios below the recommended threshold (<0.85) [21,22,36,37], thereby minimizing the potential impact of anthropometric and metabolic factors on fertility outcomes. Lifestyle behaviors are recognized determinants of reproductive function, and modifiable factors such as physical activity and body composition may influence ART outcomes. The similarity between groups in these parameters reduces the likelihood that baseline lifestyle-related confounding influenced the findings. Although moderate physical activity has been associated with improved reproductive and psychological parameters, the predominantly inactive status observed in both groups suggests that physical activity was unlikely to differentially affect outcomes in this cohort. Future studies integrating structured dietary and physical activity interventions may further clarify their combined impact on ART success.

The Mediterranean diet is widely recognized for its health benefits, including potential effects on fertility [3,12,13,14,32,33]. Most studies assessing adherence in ART populations rely on questionnaire-based tools such as food frequency questionnaires, the Mediterranean Diet Score [38], and MEDAS [39], categorizing adherence into quartiles, tertiles, or low-to-high groups [3,13,14,32,33,34]. In this study, adherence was measured using the MEDLIFE index and MDQI derived from three-day dietary records. A significant increase in MEDLIFE scores across sessions supported intervention compliance, aligning with prior methodologies, though variability in tools and cultural factors limits comparability across studies.

Energy intake in both groups (~1600–1900 kcal/day) met individual needs without restriction. Macronutrient distribution in the intervention group matched Mediterranean diet targets: protein (16–17%, 1.04 g/kg/day), carbohydrates (45–48%), and fat (35–38%), consistent with accepted ranges [40,41]. While fat intake slightly exceeded targets, it remained within Mediterranean norms (35–45%). Despite the absence of strict energy restriction, no significant weight gain was observed during the 12-week period, suggesting that the dietary pattern was metabolically balanced. Although energy intake was comparable, minor variations in caloric balance or dietary quality could still influence insulin sensitivity, inflammatory tone, and follicular metabolic environment, representing potential metabolic confounders.

Prior studies have shown similar energy, protein, and carbohydrate intake in women with infertility, with higher fat intake attributed to Mediterranean dietary patterns [13,21]. These results suggest that participants achieved macronutrient intake consistent with Mediterranean diet principles, supporting dietary fidelity and intervention validity. Similar macronutrient patterns have also been observed in preconception and ART populations adhering to Mediterranean-like or pro-fertility dietary indices. Thus, the macronutrient composition achieved in the present intervention appears comparable to ranges previously reported in reproductive health research, supporting the nutritional adequacy of the dietary model applied.

Dietary lipids affect serum and ovarian fatty acid profiles, potentially influencing oocyte and embryo development [32]. The intervention group demonstrated a modest decline in the ω-6/ω-3 ratio. Although not statistically significant, this directional change aligned with Mediterranean dietary patterns. These modest shifts in fatty acid composition, even if statistically non-significant, may be biologically relevant, given the known role of ω-3 and ω-6 balance in inflammatory modulation and oxidative regulation. From a mechanistic perspective, long-chain ω-3 PUFAs such as EPA and DHA can be incorporated into granulosa and oocyte membrane phospholipids, altering membrane fluidity, lipid raft organization, and receptor-mediated signaling. Through competition with arachidonic acid (ARA) for cyclooxygenase and lipoxygenase enzymes, EPA may shift eicosanoid production toward less pro-inflammatory 3-series prostaglandins. Given that controlled ovarian stimulation is associated with increased oxidative and inflammatory activity, modulation of redox balance and inflammatory tone within the follicular microenvironment represents a biologically plausible pathway linking fatty acid quality to oocyte maturation. However, these mechanisms were not directly measured in the present study and remain speculative.

At baseline, the intervention group had lower serum ARA and higher LA/ALA ratios than the control group. While no significant post-intervention differences were found between groups, the intervention group showed a significant decrease in serum LA and LA/ALA ratio, alongside a non-significant increase in ALA, suggesting a shift toward a more favorable fatty acid profile. Similarly, follicular fluid ALA levels were higher and the LA/ALA ratio was lower in the intervention group, aligning with dietary intake patterns. These patterns suggest that dietary fatty acid modifications were partially reflected in circulating and follicular lipid profiles, supporting the responsiveness of essential fatty acids to dietary modulation. Given the complex and stage-dependent metabolic requirements of follicular development and embryo implantation, such shifts may have biological relevance, although their direct impact on clinical outcomes remains uncertain. Previous studies have reported heterogeneous associations between ALA, LA/ALA ratios, and pregnancy outcomes, underscoring the need for cautious interpretation. Larger, well-powered randomized studies are required to determine whether these biochemical changes translate into meaningful improvements in ART success.

Although dietary fatty acid intake did not differ significantly between groups or across time points, biologically meaningful shifts were observed in serum and follicular fluid fatty acid profiles. These changes occurred despite the absence of statistically significant differences in dietary intake estimated from food records, likely reflecting that Mediterranean diet adherence was assessed using a composite score rather than strict quantitative fatty acid monitoring. Therefore, full dietary exposure may not have been completely captured.

Serum EPA and total EPA + DHA levels were initially lower in the intervention group and declined further by study end. No significant increase was observed despite reported dietary adherence. This may partly reflect higher baseline values in the control group and limited direct EPA/DHA intake. Given that endogenous conversion of ALA to long-chain ω-3 fatty acids is minimal [42], insufficient fatty fish consumption may explain the lack of serum EPA/DHA elevation. Furthermore, inter-individual variability in fatty acid absorption, metabolism, and incorporation into circulating lipids [43] likely contributed to heterogeneous biochemical responses. Thus, circulating fatty acid levels should not be interpreted as a direct or linear reflection of dietary intake.

In follicular fluid, the intervention group exhibited significantly lower EPA + DHA, total ω-6 fatty acids, LA/ALA ratio, and ω-6/ω-3 ratio compared to controls (*p* < 0.05). While these findings suggest local shifts in ovarian lipid milieu, they must be interpreted cautiously. Fatty acid concentrations were expressed as relative percentages, and fluctuations in plasma lipid fractions or volume distribution over time may influence proportional representation. Moreover, given the limited sample size—particularly for follicular fluid analyses—these findings should be regarded as exploratory and hypothesis-generating rather than confirmatory.

The observed reductions in serum and follicular fluid ω-6/ω-3 ratios align directionally with expected Mediterranean dietary effects. However, the absence of consistent EPA/DHA elevation indicates that achieving biologically meaningful increases may require structured monitoring of specific fatty fish intake or supplementation, as suggested in prior studies [18,44,45]. Variability in EPA/DHA content across fish species [46] further highlights the importance of precise dietary specification [47].

Overall, while certain favorable trends were detected in systemic and follicular fatty acid balance, the clinical implications remain uncertain. Future adequately powered randomized controlled trials incorporating quantitative fatty acid monitoring, standardized dietary implementation, and predefined reproductive endpoints are necessary to determine whether these biochemical shifts translate into meaningful ART outcomes.

In this study, clinical pregnancy occurred in 3 participants in the intervention group and 1 participant in the control group. Two live births were recorded, both in the intervention group. These pregnancy rates were lower than those reported in previous studies (27.6–50.9%) [13,48], possibly due to variations in sample size, characteristics, and study design. Research on ω-3 fatty acids and reproductive outcomes has yielded inconsistent results, ranging from positive [49,50] to null or even adverse effects [51,52], largely influenced by dosage, source, and exposure duration [49]. Although pregnant participants in the intervention group reported higher total ω-3 intake, both serum and follicular fluid EPA + DHA levels were unexpectedly lower. This discrepancy may reflect individual variability in fatty acid absorption and metabolism, the limited number of follicular samples, or external dietary factors such as seasonal fish consumption. These findings highlight the complexity of linking self-reported intake to biological incorporation and suggest that diet–biomarker relationships may not always be linear. Therefore, our results should be interpreted cautiously and require confirmation in larger controlled trials. This aligns with earlier findings linking ω-3 PUFA intake to improved implantation and pregnancy outcomes [16,48,53], though geographic and cohort-based differences exist. Studies evaluating Mediterranean diet adherence and IVF outcomes have shown mixed results. Some reported positive associations with clinical pregnancy or live birth [12,32,33], while others found no significant relationship [3,13]. Variability in age, adherence level, and study design likely contribute to these inconsistencies. Further standardized, controlled dietary intervention studies are needed to clarify the diet’s impact on ART outcomes. Importantly, the observed clinical pregnancies and live births in the intervention group, although numerically higher, should be interpreted as exploratory findings given the small number of events and limited statistical power. The present results do not allow causal inference regarding dietary effects on ART success. Well-designed, adequately powered randomized controlled trials with predefined reproductive endpoints are required to confirm whether dietary modulation translates into clinically meaningful improvements in fertility outcomes. Future studies should also incorporate standardized dietary monitoring and quantitative biomarker assessment to better clarify diet–reproduction interactions.

The observed correlations between specific diet quality indices and IVF parameters should also be interpreted within an exploratory framework. While MDQI scores showed positive associations with MII oocyte number and pronuclei in the intervention group, overall Mediterranean diet adherence (MEDLIFE) was not consistently related to IVF parameters, and dietary fatty acid ratios were not significantly associated with clinical outcomes. Moreover, the heterogeneous direction of serum fatty acid correlations—both positive (e.g., total ω-3 with MII oocytes) and inverse (e.g., EPA and DHA with oocyte parameters)—suggests complex metabolic regulation rather than a direct causal dietary effect. Given the limited sample size and multiple correlation analyses performed, these findings may reflect statistical variability rather than stable biological relationships. Similar inconsistencies have been reported in prior reproductive nutrition studies, particularly those with small intervention samples or observational designs, where associations between fatty acids and oocyte quality were not uniformly replicated. Therefore, these correlations should be considered hypothesis-generating and require confirmation in adequately powered randomized trials with predefined reproductive endpoints.

A human study reported an inverse correlation between follicular fluid LA levels and metaphase II (MII) oocyte count [15], while others have shown that elevated LA and ARA may impair oocyte competence without affecting pregnancy rates [54]. Matorras et al. [55] found that ALA and ARA levels decline during maturation, with reduced ARA:DHA and ARA:LA ratios by the MII stage, suggesting a less inflammatory profile is favorable for mature oocytes. Mechanistically, LA serves as a precursor of arachidonic acid (ARA), which is metabolized through cyclooxygenase and lipoxygenase pathways into bioactive eicosanoids. Excessive ω-6-derived eicosanoid production may increase local inflammatory tone within the follicular microenvironment, potentially affecting cumulus–oocyte communication, mitochondrial function, and meiotic progression. A reduction in ARA:DHA and ARA:LA ratios toward the MII stage may therefore reflect a shift toward a less pro-inflammatory lipid milieu that supports cytoplasmic and nuclear maturation. In the present study, serum LA/ALA ratio showed a moderate inverse correlation with oocyte maturation rate and a positive correlation with pronucleus count, while serum ALA was inversely associated with maturation rate. These findings align with the hypothesis that inflammation is downregulated during oocyte maturation. However, the inverse association between ALA and maturation suggests a complex metabolic interplay requiring further study. No associations were observed between follicular fluid ALA or LA/ALA ratios and IVF or pregnancy outcomes. Differences in study designs and metabolic demands across treatment stages may explain discrepancies. Additionally, the absence of standardized cut-off values for serum and follicular fluid fatty acids limits interpretation. Overall, findings support the importance of balanced fatty acid intake and warrant further mechanistic research.

In the intervention group, no significant associations were observed between serum or follicular fluid levels of total ω-6, total ω-3, or the ω-6/ω-3 ratio and IVF or pregnancy outcomes. Similarly, Jungheim et al. [53] and Hammiche et al. [56] reported no associations between serum ω-3 PUFA levels and ART parameters. However, in the control group, serum ω-3 PUFAs were negatively associated with MII oocyte count and pronucleus number, while follicular fluid ω-3 levels correlated negatively with total oocyte count. Although some studies suggest beneficial effects of higher ω-3 levels—particularly EPA—on ART outcomes [15,16,17,18], the present findings contrast with those of Chiu et al. [16], who reported increased pregnancy and live birth rates with higher serum ω-3, especially EPA. Similarly, Kermack et al. [17,18] found improved follicular fluid composition and embryo quality following EPA + DHA supplementation. In this study, no associations were found between EPA, DHA, or EPA + DHA levels and IVF parameters in the intervention group. In the control group, EPA and DHA levels were inversely associated with total oocyte, MII oocyte, and pronucleus counts, but not with maturation or fertilization rates. These findings highlight the complexity of ω-3 fatty acid effects and suggest that outcomes may depend on dosage, timing, and individual variability. From a biological perspective, ω-3 PUFAs such as EPA and DHA are incorporated into cell membrane phospholipids, altering membrane fluidity and lipid raft organization, thereby modulating receptor signaling and intracellular pathways. They also compete with ARA for cyclooxygenase enzymes, shifting prostaglandin synthesis toward less inflammatory 3-series derivatives. However, because highly unsaturated fatty acids are more susceptible to lipid peroxidation, excessive or unbalanced accumulation may increase oxidative stress within the follicular environment, potentially impairing mitochondrial ATP production and meiotic spindle stability. This dual role may explain the inconsistent associations observed across studies.

Although changes in serum and follicular fluid EPA and DHA levels were linked to IVF parameters, these associations did not extend to clinical pregnancy outcomes. This aligns with prior studies that found no significant relationship between ω-3 fatty acids and pregnancy success [15,16,53,54]. While this study adds to the limited evidence in infertile populations, it also underscores the need for further research on the reproductive effects of ω-3 fatty acids. Fatty acid levels were evaluated in relation to key oocyte parameters—total count, MII oocytes, pronucleus number, maturation, and fertilization rates. The results showed partial agreement with previous findings [12,16] and notable differences from others [53,57], particularly in the lack of correlation with pregnancy or live birth outcomes. These findings support the notion that oocyte quality indicators alone may not reliably predict clinical success, reinforcing the complexity of factors influencing reproductive outcomes. Importantly, oocyte maturation and early embryonic parameters represent only the initial stages of a multifactorial reproductive cascade. Implantation and clinical pregnancy additionally depend on endometrial receptivity, immune modulation, angiogenesis, and embryo–maternal signaling. Fatty acids may exert differential effects at each of these stages through regulation of PPAR signaling, steroidogenesis, cytokine balance, and oxidative homeostasis. Therefore, improvements or alterations at the oocyte level may not necessarily translate into measurable differences in pregnancy or live birth outcomes.

At the end of the intervention, a significant positive correlation was found only between MEDLIFE scores and total serum ω-6 fatty acid levels, while no associations were observed between Mediterranean diet adherence and follicular fluid fatty acid levels. Previous research has shown that serum fatty acids can be reflected in follicular fluid, even at low concentrations [58,59,60]. Mirabi et al. [48] similarly reported correlations between serum and follicular fluid levels of essential fatty acids such as ALA and LA. In the current study, moderate correlations were observed between serum and follicular fluid ω-6, ω-3, and EPA + DHA levels, supporting their metabolic link. Additionally, significant associations were found between the follicular fluid LA/ALA ratio and dietary LA/ALA and ω-6/ω-3 ratios. These findings, independent of the intervention, suggest that dietary intake may influence the follicular environment, contributing to the limited literature in this area. Given that ω-6 and ω-3 fatty acids act via enzymatic pathways (COX, LOX, CYP) and signal through GPCRs and PPARs [61], maintaining a balanced dietary fatty acid profile is critical for modulating reproductive metabolism. Mechanistically, circulating fatty acids can be incorporated into follicular phospholipid membranes, thereby influencing membrane fluidity, lipid raft organization, and receptor-mediated signaling within granulosa and cumulus cells. Through competitive metabolism of arachidonic acid and EPA via cyclooxygenase and lipoxygenase pathways, the ω-6/ω-3 balance may shift the production of bioactive lipid mediators toward either pro-inflammatory (2-series prostaglandins, leukotrienes) or less inflammatory 3-series derivatives. In addition, activation of PPAR isoforms by long-chain PUFAs may regulate genes involved in steroidogenesis, β-oxidation, mitochondrial function, and oxidative stress control. These pathways provide a biological framework through which dietary fatty acid patterns may shape the follicular microenvironment and potentially influence oocyte developmental competence.

These findings are biologically plausible, as essential fatty acids such as LA and ALA cannot be synthesized de novo and depend on dietary intake. However, the endogenous conversion of ALA to long-chain ω-3 fatty acids (EPA and DHA) is relatively limited in humans, with reported conversion rates generally below 5–10%. Therefore, follicular EPA and DHA levels likely reflect not only direct dietary intake but also individual variability in desaturase and elongase enzyme activity. This metabolic interplay may partly explain inter-individual differences in follicular fatty acid composition despite similar dietary patterns.

Moreover, because fatty acids were expressed as relative percentages rather than absolute concentrations, changes in circulating lipid fractions or plasma volume during controlled ovarian stimulation may influence proportional values. Hemodilution, altered lipoprotein transport, or shifts in triglyceride and phospholipid distribution could modify percentage-based fatty acid profiles without necessarily indicating true changes in absolute tissue exposure. Thus, both dietary intake and metabolic processing, together with methodological considerations related to relative quantification, should be considered when interpreting these associations.

### 4.1. Strengths, Limitations, and Weaknesses

#### 4.1.1. Strengths

A major strength of this study is the individualized Mediterranean diet-based intervention tailored to participants’ nutritional requirements rather than relying solely on fixed portion-based recommendations. While Mediterranean diet guidelines are typically expressed as standardized daily or weekly servings, this approach accounted for individual energy and nutrient needs, thereby providing methodological innovation with potential clinical relevance.

The validation of the Turkish version of the Mediterranean Lifestyle Index during the study period represents an additional contribution, enhancing methodological rigor and expanding validated assessment tools available in Turkish.

Fatty acid analyses in both serum and follicular fluid were performed using gas chromatography (GC-FID), improving analytical precision. Given that circulating long-chain ω-3 fatty acids have been shown to reflect habitual intake, the collection of repeated serum measurements in the intervention group strengthens the biomarker-based evaluation.

The use of follicular fluid, a biologically relevant reproductive matrix, and its comparative analysis with serum fatty acid profiles enhances the translational value of the findings.

#### 4.1.2. Limitations

The primary limitation of this study is the relatively small sample size, particularly the limited number of follicular fluid samples and clinical pregnancies. This reduced statistical power and limited the ability to detect differences in clinical endpoints. Because ART outcomes were not powered as primary endpoints, these findings should be interpreted as exploratory and hypothesis-generating. Future trials with sample size calculations based specifically on omega-3 levels and clinically meaningful reproductive outcomes may provide more robust conclusions.

The inclusion of heterogeneous infertility diagnoses and both IUI and IVF protocols may have introduced confounding. Protocol transitions during follow-up further reduced cohort homogeneity. Future studies should consider more homogeneous diagnostic groups and standardized ART protocols.

The study employed a non-randomized, sequential recruitment design. This approach may introduce potential selection bias and time-related confounding, thereby limiting causal inference. However, the design was driven by the intrinsic short duration of IVF protocols (12–15 days) relative to the 12-week dietary intervention, reflecting feasibility considerations rather than arbitrary allocation.

The ≥50% MEDLIFE adherence cut-off may limit comparability with other studies due to variability in adherence categorization.

As it was conducted during the COVID-19 pandemic, the study faced reduced referrals, treatment cancelations, staff turnover, and operational challenges. Seasonal dietary variations may also represent a potential confounder.

Follicular fluid could only be collected from patients undergoing IVF/ICSI with oocyte retrieval, leading to missing samples in some intervention participants. The short and complex nature of ART procedures, along with pregnancy occurrence, psychological distress, or treatment center changes, contributed to attrition.

Given the limited number of clinical events, clinical outcomes were interpreted descriptively and should be considered exploratory.

Clinical trial registration was completed retrospectively. Although ethical approval and predefined outcomes were established prior to recruitment and no post hoc modifications were made, this should be considered when interpreting the findings.

Overall, the present results should be regarded as preliminary and hypothesis-generating. Larger, randomized, parallel-group studies with homogeneous cohorts and adequately powered clinical endpoints are required to confirm these findings.

## 5. Conclusions

This study evaluated a 12-week Mediterranean diet-based intervention in women undergoing ART during the preconception period, examining its associations with serum and follicular fluid fatty acid profiles, ART outcomes, and implantation findings. Although favorable trends were observed in certain fatty acid profiles and ART-related parameters, the findings do not allow for definitive conclusions regarding clinical impact. In particular, analyses related to ART outcomes and clinical endpoints should be considered exploratory in nature given the study design and sample size.

The partial reflection of dietary fatty acid patterns in biological samples may suggest a complex relationship between dietary intake, metabolic incorporation, and reproductive outcomes. However, the current results represent preliminary evidence and are insufficient to support clinical dietary recommendations.

Larger, well-designed randomized controlled trials in which ART and clinical endpoints are defined as primary outcomes are needed. Studies with increased sample sizes and more homogeneous patient groups may help clarify the potential role of Mediterranean diet adherence in reproductive outcomes.

## Figures and Tables

**Table 1:** Recommended Daily and Weekly Servings According to the Mediterranean Lifestyle (MEDLIFE) Index.

Daily Recommendations	Weekly Recommendations
Low-fat dairy products: 2 servings/day Skim milk (1 serving = 1 glass or 200 mL)	Fish/Seafood: ≥2 servings/week White or oily fish (1 serving = 100–150 g), canned fish (1 serving = 1 can or 50 g), seafood (1 serving = 200 g)
Nuts and Olives: 1–2 servings/day Walnuts, almonds, hazelnuts (1 serving = 1 handful or 30 g); olives (1 serving = 10 pieces)	Red Meat: <2 servings/week Beef, pork, lamb (1 serving = 100–150 g)
Fruits: 3–6 servings/day All types of fruits and freshly squeezed fruit juices (1 serving = 150–200 g)	Processed Meat: ≤1 serving/week Ham (1 serving = 1 slice or 30 g), sausage, salami, pastrami (1 serving = 50 g), hamburger (1 serving = 1 piece), organ meats such as liver (1 serving = 100–150 g)
Vegetables: ≥2 servings/day All vegetables except potatoes (1 serving = 150–200 g)	White Meat: 2 servings/week (1 serving = 100–150 g)
Olive Oil: ≥3 tablespoons/day Olive oil, extra virgin olive oil (1 serving = 1 tablespoon)	Legumes: ≥2 servings/week Lentils, beans, peas, chickpeas (1 serving = 1 plate or 150 g)
Grains: 3–6 servings/day White and whole grain bread (1 serving = 40 g), grains and their derivatives (1 serving = 1 dinner plate)	Eggs: 2–4 servings/week Egg (1 serving = 1 egg)
Water: 6–8 servings/day (1 serving = 1 glass of water)	Potatoes: ≤3 servings/week Baked/boiled potatoes, French fries (1 serving = 150–200 g)
	Sweets: ≤2 servings/week Sugar/candy (1 serving = 1 piece or 50 g), chocolate (1 serving = 30 g), biscuits/cookies (1 serving = 4–6 pieces)

**Table 2:** Descriptive Characteristics of the Participants.

	Intervention Group (*n* = 16)		Control Group (*n* = 16)		
**History**	$\bar{x}$ ± SS (Min–Max)	Median (IQR)	$\bar{x}$ ± SS (Min–Max)	Median (IQR)	*p*
Age (years)	27.6 ± 4.69 (19.0–36.0)	27.5 (7.0)	29.8 ± 4.81 (23.0–40.0)	29.0 (6.0)	0.224
Duration of Marriage (years)	3.6 ± 2.27 (1.0–10.0)	3.3 (2.8)	4.6 ± 3.77 (1.0–16.0)	3.5 (3.9)	0.616
Duration of Infertility (years)	2.6 ± 1.53 (0.6–5.5)	2.0 (2.3)	3.1 ± 1.89 (1.0–7.0)	2.3 (3.1)	0.381
Age at Menarche (years)	14.0 ± 1.04 (12.0–16.0)	13.9 (0.8)	13.7 ± 0.92 (12.0–15.0)	13.8 (1.0)	0.515
Menstrual Cycle Regularity (days)	31.2 ± 3.82 (27.0–40.0)	30.0 (0.0)	31.5 ± 5.84 (26.0–50.0)	30.7 (1.8)	0.897
Number of IUI Attempts	1.6 ± 1.00 (1.0–2.0)	2.0 (1.0)	1.9 ± 0.25 (1.0–2.0)	2.0 (0.1)	0.323
Number of IVF/ICSI Cycles	1.6 ± 0.50 (1.0–2.0)	2.0 (1.0)	1.8 ± 0.1 (1.0–2.0)	2.0 (0.1)	0.780
AFC (count)	13.5 ± 9.28 (0.0–30.0)	14.0 (14.8)	13.9 ± 8.69 (0.0–30.0)	13.0 (15.5)	0.715
Right Ovary AFC (count)	8.1 ± 3.61 (3.0–15.0)	8.0 (7.0)	7.6 ± 3.63 (2.0–12.0)	7.5 (7.8)	0.780
Left Ovary AFC (count)	7.6 ± 3.49 (2.0–12.0)	8.0 (5.0)	7.0 ± 3.93 (0.0–12.0)	6.0 (8.0)	0.724
Physical Activity Levels					
IPAQ-SF (Sitting Time, hours/day)	7.3 ± 2.52 (4.6–12.3)	6.6 (3.2)	7.0 ± 2.85 (4.0–11.6)	6.4 (3.1)	0.516
IPAQ-SF (Total MET-minutes/week)	676.6 ± 747.63 (0.0–3126.0)	594.0 (639.8)	496.0 ± 537.52 (0.0–1386.0)	297.0 (940.5)	0.491
Anthropometric Measurements					
BMİ (kg/m^2^)	23.5 ± 3.30 (18.5–29.4)	22.7 (5.3)	23.2 ± 2.96 (18.3–27.6)	23.1 (5.4)	0.734
Waist-to-Hip Ratio	0.8 ± 0.11 (0.6–0.9)	0.7 (0.2)	0.7 ± 0.07 (0.6–0.9)	0.7 (0.11)	0.113
Body Fat Percentage (%)	28.6 ± 7.94 (15.2–42.6)	29.2 (11.5)	29.2 ± 7.33 (15.0–39.1)	29.2 (11.1)	0.734
Fat-Free Mass (kg)	40.7 ± 3.07 (34.2–47.6)	41.1 (2.7)	40.2 ± 2.14 (36.3–45.2)	39.9 (3.0)	0.534

Mann–Whitney U test, *p* < 0.05. IUI: Intrauterine Insemination; IVF/ICSI: In Vitro Fertilization/Intracytoplasmic Sperm Injection; AFC: Antral Follicle Count; IPAQ-SF: International Physical Activity Questionnaire–Short Form; IQR: interquartile range.

**Table 3:** Women’s Diet Adherence (MEDLIFE and MDQI Scores and Dietary Intake Findings).

	Intervention Group (First) (*n* = 16)		Intervention Group (Second) (*n* = 16)		Intervention Group (Third) (*n* = 16)		Intervention Group (Last) (*n* = 16)		Control Group (*n* = 16)	
	**$\bar{x}$ ± SS** **(Min–Max)**	**Median** **(IQR)**	**$\bar{x}$ ± SS** **(Min–Max)**	**Median** **(IQR)**	**$\bar{x}$ ± SS** **(Min–Max)**	**Median** **(IQR)**	**$\bar{x}$ ± SS** **(Min–Max)**	**Median** **(IQR)**	**$\bar{x}$ ± SS** **(Min–Max)**	**Median** **(IQR)**
MEDLIFE (score)	13.7 ± 3.86 (7.0–21.0)	14.5 (4.8)	16.8 ± 1.73 (14.0–20.0)	16.5 (2.8)	16.4 ± 2.10 (12.0–20.0)	16.0 (3.0)	15.9 ± 2.47 (13.0–27.0)	15.0 (4.0)	11.0 ± 3.68 (6.0–17.0)	12.0 (6.0)
	*p*^1^ = 0.225; *p*^2^ = 0.007 *; *p*^3^ = 0.024 *; *p*^4^ = 0.007 *									
MDQI (score)	5.6 ± 1.37 (3.0–8.0)	5.0 (2.0)	5.1 ± 1.29 (3.0–7.0)	5.0 (2.0)	4.6 ± 1.93 (2.0–8.0)	4.0 (3.0)	5.1 ± 1.44 (3.0–7.0)	5.0 (2.0)	6.3 ± 1.74 (3.0–9.0)	6.5 (2.8)
	*p*^1^ = 0.195; *p*^2^ = 0.328; *p*^3^ = 0.369; *p*^4^ = 0.046 *									
Energy (kcal/day)	1979.0 ± 293.0 (1517.0–2384.2)	2089.0 (523.5)	1894.3 ± 424.2 (1320.5–2640.4)	1884.7 (747.3)	2000.8 ± 347.7 (1503.8–2785.4)	1880.1 (480.8)	2045.7 ± 364.0 (1223.2–2495.9)	2121.4 (515.6)	1855.5 ± 372.7 (1212.3–2630.7)	1794.3 (409.7)
*p*^1^ = 0.214; *p*^2^ = 0.631; *p*^3^ = 0.541; *p*^4^ = 0.083										
Protein (% of daily energy)	13.9 ± 2.6 (10.0–20.0)	14.0 (4.0)	13.1 ± 1.7 (10.0–16.0)	13.5 (2.0)	13.4 ± 2.50 (10.0–18.0)	12.5 (5.0)	13.3 ± 1.80 (11.0–19.0)	13.0 (2.0)	13.6 ± 1.5 (10.0–15.0)	14.0 (2.0)
*p*^1^ = 0.818; *p*^2^ = 0.872; *p*^3^ = 0.344; *p*^4^ = 0.183										
Protein (g/day)	67.3 ± 14.71 (41.5–88.9)	68.8 (22.8)	61.8 ± 18.06 (37.0–91.2)	64.6 (31.4)	65.0 ± 11.55 (44.9–83.1)	64.8 (14.5)	66.7 ± 14.84 (37.0–99.5)	68.6 (17.5)	61.2 ± 12.34 (39.4–88.9)	60.6 (12.4)
*p*^1^ = 0.163; *p*^2^ = 0.700; *p*^3^ = 0.877; *p*^4^ = 0.214										
Plant-Derived Protein (g)	36.8 ± 5.27 (28.1–45.2)	38.2 (8.1)	36.5 ± 11.71 (21.7–56.6)	37.1 (19.5)	39.9 ± 11.59 (19.6–63.6)	38.5 (13.3)	40.5 ± 10.93 (18.9–62.3)	39.0 (17.0)	34.7 ± 9.4 (23.6–60.6)	33.2 (12.7)
*p*^1^ = 0.169; *p*^2^ = 0.960; *p*^3^ = 0.169; *p*^4^ = 0.260										
Animal-Derived Protein (g)	30.8 ± 11.47 (7.6–48.5)	31.4 (19.4)	25.3 ± 10.92) (7.0–47.6)	23.7 (15.8)	25.1 ± 11.61 (5.3–41.7)	26.2 (21.2)	26.2 ± 9.99 (12.6–46.0)	23.2 (14.2)	26.5 ± 9.85 (5.4–44.6)	26.9 (16.1)
*p*^1^ = 0.214; *p*^2^ = 0.080; *p*^3^ = 0.148; *p*^4^ = 0.821										
Carbohydrate (% of daily energy)	44.5 ± 5.0 (35.0–55.0)	44.0 (5.0)	45.2 ± 6.90 (35.0–59.0)	42.0 (9.0)	45.8 ± 8.5 (35.0–68.0)	40.0 (7.0)	44.2 ± 5.8 (35.0–53.0)	43.0 (11.0)	41.8 ± 6.5 (32.0–55.0)	41.5 (9.0)
*p*^1^ = 0.096; *p*^2^ = 0.617; *p*^3^ = 0.293; *p*^4^ = 0.257										
Fiber (g)	23.0 ± 6.9 (13.7–31.5)	21.8 (7.1)	24.9 ± 7.1 (12.6–37.2)	23.3 (10.7)	26.5 ± 9.8 (13.1–49.5)	23.9 (9.0)	26.5 ± 7.13 (17.5–41.9)	25.6 (12.2)	22.5 ± 6.73 (12.7–36.1)	21.1 (10.9)
*p*^1^ = 0.546; *p*^2^ = 0.428; *p*^3^ = 0.155; *p*^4^ = 0.200										
Soluble fiber (g)	7.6 ± 2.16 (4.0–10.8)	7.5 (84.1)	7.9 ± 2.73 (3.1–12.2)	7.4 (4.4)	8.6 ± 3.81 (3.0–16.8)	7.4 (1.7)	8.7 ± 2.33 (5.8–12.8)	8.1 (3.9)	6.8 ± 2.0 (3.6–10.8)	6.5 (2.2)
*p*^1^ = 0.274; *p*^2^ = 0.248; *p*^3^ = 0.163; *p*^4^ = 0.019										
Insoluble fiber (g)	14.6 ± 2.85 (8.4–18.5)	14.6 (3.8)	16.2 ± 4.06 (8.1–22.2)	15.0 (6.3)	17.5 ± 6.02 (9.6–31.0)	15.2 (4.3)	17.1 ± 4.41 (10.5–25.3)	17.0 (7.4)	14.9 ± 5.1 (7.6–25.5)	14.6 (7.7)
*p*^1^ = 0.880; *p*^2^ = 0.272; *p*^3^ = 0.063; *p*^4^ = 0.258										
Fat (% of daily energy)	41.4 ± 4.8 (33.0–49.0)	42.0 (6.0)	41.4 ± 6.7 (27.0–50.0)	43.5 (10.0)	40.6 ± 7.9 (20.0–53.0)	43.5 (12.0)	42.6 ± 5.7 (33.0–51.0)	43.5 (11.0)	44.7 ± 7.0 (30.0–55.0)	46.5 (8.0)
*p*^1^ = 0.125; *p*^2^ = 0.867; *p*^3^ = 0.531; *p*^4^ = 0.273										
SFA (%)	13.3 ± 1.72 (10.4–16.3)	13.6 (2.7)	12.9 ± 2.01 (9.9–18.8)	13.1 (2.1)	12.3 ± 3.06 (7.3–18.8)	12.3 (3.3)	12.4 ± 1.38 (9.4–14.8)	12.5 (1.4)	13.6 ± 3.06 (7.0–18.6)	13.9 (3.6)
*p*^1^ = 0.515; *p*^2^ = 0.318; *p*^3^ = 0.079; *p*^4^ = 0.128										
MUFA (%)	17.1 ± 3.33 (12.1–23.8)	17.1 (6.0)	18.6 ± 4.89 (11.7–27.8)	18.0 (8.3)	17.4 ± 3.81 (10.4–26.8)	17.2 (5.0)	17.8 ± 3.26 (10.8–22.8)	18.1 (4.2)	15.1 ± 3.36 (9.3–23.5)	14.6 (4.0)
*p*^1^ = 0.094; *p*^2^ = 0.665; *p*^3^ = 0.679; *p*^4^ = 0.035										
PUFA (%)	11.9 ± 2.83 (6.0–17.8)	10.4 (2.7)	10.1 ± 3.16 (5.2–16.8)	10.1 (4.7)	13.9 ± 3.08 (8.9–19.8)	13.0 (5.6)	11.7 ± 3.03 (6.8–18.8)	10.9 (4.3)	10.1 ± 2.18 (5.5–13.9)	10.5 (2.7)
*p*^1^ = 0.590; *p*^2^ = 0.068; *p*^3^ = 0.796; *p*^4^ = 0.270										
Cholesterol (mg)	284.0 ± 74.49 (173.2–391.3)	277.6 (140.9)	242.6 ± 81.15 (69.9–398.0)	252.0 (100.4)	267.2 ± 71.91 (121.2–377.9)	275.7 (81.5)	280.4 ± 75.46 (147.3–389.6)	282.6 (111.4)	284.1 ± 110.0 (114.1–551.7)	289.8 (169.8)
*p*^1^ = 0.678; *p*^2^ = 0.508; *p*^3^ = 0.959; *p*^4^ = 0.910										
ω-3 fatty acids (g)	2.3 ± 1.05 (0.8–4.2)	2.4 (1.8)	2.8 ± 1.40 (0.7–6.0)	2.5 (1.4)	3.7 ± 1.45 (0.9–6.0)	4.0 (2.3)	2.9 ± 1.51 (0.7–5.5)	3.2 (2.9)	2.03 ± 1.2 (0.6–4.7)	1.7 (1.5)
*p*^1^ = 0.336; *p*^2^ = 0.095; *p*^3^ = 0.379; *p*^4^ = 0.132										
ω-6 fatty acids (g)	21.5 ± 5.38 (12.7–33.6)	22.4 (8.2)	20.2 ± 7.85 (10.2–34.4)	19.4 (14.6)	26.0 ± 7.75 (14.3–45.6)	24.9 (10.4)	22.6 ± 6.76 (12.2–31.8)	23.5 (14.2)	18.7 ± 5.8 (10.3–31.2)	17.1 (7.7)
*p*^1^ = 0.152; *p*^2^ = 0.264; *p*^3^ = 0.569; *p*^4^ = 0.113										
EPA + DHA (g)	0.3 ± 0.55 (0.1–2.3)	0.1 (0.3)	0.3 ± 0.56 (0.1–2.0)	0.1 (0.4)	0.2 ± 0.35 (0.1–1.4)	0.1 (0.4)	0.1 ± 0.11 (0.1–0.5)	0.1 (0.1)	0.2 ± 0.29 (0.1–1.0)	0.1 (0.1)
*p*^1^ = 0.341; *p*^2^ = 0.741; *p*^3^ = 0.120; *p*^4^ = 0.809										

*p*^1^: Baseline comparison between control and intervention groups, Mann–Whitney U test, * *p* < 0.05. *p*^2^: Within-group comparison across all time points in the intervention group, Friedman, * *p* < 0.05. *p*^3^: Pre- and post-intervention comparison within the intervention group, Wilcoxon, * *p* < 0.05. *p*^4^: Post-intervention comparison between intervention and control groups, Mann–Whitney U test, * *p* < 0.05. MEDLIFE: The Mediterranean Lifestyle index; MDQI: Mediterranean Diet Quality Index. SFA: Saturated Fatty Acids; MUFA: Monounsaturated Fatty Acids; PUFA: Polyunsaturated Fatty Acids.

**Table 4:** Fatty Acid Levels Calculated from Women’s Dietary Intake Records and Analyzed in Serum and Follicular Fluid.

	Intervention Group (First) (*n* = 16)		Intervention Group (Last) (*n* = 16)		Control Group (*n* = 16)				
**Dietary Fatty Acid Ratio**	**$\bar{x}$ ± SS** **(Min–Max)**	**Median** **(IQR)**	**$\bar{x}$ ± SS** **(Min–Max)**	**Median** **(IQR)**	**$\bar{x}$ ± SS** **(Min–Max)**	**Median** **(IQR)**	***p*** **^1^**	***p*** **^2^**	***p*** **^3^**
ω-6 fatty acids (%)	23.7 ± 4.92 (16.0–36.2)	22.1 (5.4)	23.9 ± 4.09 (16.7–30.9)	23.9 (6.1)	23.7 ± 6.58 (14.6–41.9)	22.4 (5.4)	0.867	0.468	0.796
ω-3 fatty acids (%)	2.6 ± 1.17 (1.0–4.9)	2.7 (1.8)	3.0 ± 1.37 (1.0–5.6)	3.1 (2.4)	2.4 ± 1.16 (1.2–4.8)	2.1 (1.2)	0.539	0.210	0.301
ω-6/ω-3	11.0 ± 5.55 (5.9–24.8)	8.1 (8.4)	9.6 ± 4.58 (4.6–19.3)	7.6 (7.3)	11.7 ± 6.79 (4.6–32.5)	10.5 (6.4)	0.809	0.270	0.501
LA/ALA	13.2 ± 6.78 (5.99–27.52)	9.7 (11.3)	10.0 ± 4.94 (4.7–20.3)	8.3 (8.0)	13.7 ± 7.92 (5.9–33.6)	11.2 (10.6)	0.956	0.196	0.070
EPA + DHA fatty acids (%)	0.3 ± 0.55 (0.0–2.3)	0.1 (0.3)	0.1 ± 0.11 (0–0.5)	0.1 (0.1)	0.2 ± 0.29 (0–1.0)	0.1 (0.1)	0.341	0.809	0.098
Serum fatty acid ratio									
ω-6 fatty acids (%)	12.9 ± 5.47 (4.4–24.7)	12.4 (7.2)	11.6 ±10.01 (4.5–21.5)	9.6 (4.8)	9.8 ± 4.39 (4.8–21.5)	9.4 (5.8)	0.055	0.925	0.088
ω-3 fatty acids (%)	9.4 ± 3.06 (5.5–15.9)	9.4 (5.2)	12.2 ±8.80 (3.5–25.6)	9.0 (7.4)	12.3 ± 3.50 (7.4–18.9)	12.3 (3.0)	0.021 *	0.187	0.326
ω-6/ω-3	1.6 ± 1.16 (0.3–4.5)	1.3 (1.3)	1.2 ±0.85 (0.3–3.1)	0.8 (1.0)	0.9 ± 0.52 (0.3–2.1)	0.7 (0.6)	0.016 *	0.366	0.163
LA/ALA	3.1 ± 2.09 (0.3–7.0)	2.7 (3.5)	1.2 ± 0.91 (0.1–3.2)	1.0 (1.2)	1.4 ± 1.49 (0.1–5.3)	0.7 (1.5)	0.026 *	0.407	0.005 *
EPA + DHA fatty acids (%)	9.6 ± 2.55 (4.2–13.3)	9.8 (4.5)	3.3 ± 0.81 (2.0–5.7)	3.2 (0)	8.8 ± 1.78 (4.9–12.6)	9.1 (0.6)	0.200	0.000	0.000 *
Follicular fluid fatty acid ratio			Intervention group (last) (*n* = 16)		Control group (*n* = 16)				
ω-6 fatty acids (%)	-	-	12.2 ± 3.38 (9.9–19.6)	11.3 (2.2)	20.5 ± 4.9 (13.2–31.0)	20.9 (6.6)	-	0.001 *	-
ω-3 fatty acids (%)	-	-	12.7 ± 13.01 (4.5–21.5)	13.0 (3.2)	12.2 ± 4.48 (5.8–16.8)	10.6 (3.6)	-	0.316	-
ω-6/ω-3	-	-	1.3 ± 1.34 (0.5–4.4)	1.0 (0.4)	1.9 ± 0.52 (0.8–2.6)	2.0 (0.7)	-	0.019 *	-
LA/ALA	-	-	0.5 ± 0.73 (0.1–2.1)	0.8 (1.0)	1.7 ± 1.29 (0.1–4.6)	1.2 (1.2)	-	0.016 *	-
EPA + DHA fatty acids (%)	-	-	5.8 ± 1.63 (3.6–7.8)	5.9 (0.1)	8.8 ± 1.76 (4.9–10.6)	7.3 (0.4)	-	0.000 *	-

*p*^1^: Comparison between the intervention group’s baseline values and those of the control group, Mann–Whitney U test, * *p* < 0.05. *p*^2^: Comparison between the post-intervention values of the intervention group and the control group, Mann–Whitney U test, * *p* < 0.05. *p*^3^: Change from baseline to post-intervention in the intervention group, Wilcoxon, * *p* < 0.05. LA/ALA: Linoleic Acid/Alpha-linolenic acid; EPA + DHA: Eikosapentaenoic acid + Docosaheksaenoic acid; %: the percentage of the specific fatty acid relative to the total fat intake from the diet.

**Table 5:** Correlation of Serum and Follicular Fluid Fatty Acid Levels with MEDLIFE and MDQI Scores.

Scores	MEDLIFE						MDQI					
	Intervention				Control		Intervention				Control	
	Before		After				Before		After			
	*p*	r	*p*	r	*p*	r	*p*	r	*p*	r	*p*	r
Serum												
ω-6 fatty acids (%)	0.488	0.187	0.036 *	0.526	0.687	0.109	0.931	−0.024	0.934	0.023	0.313	0.269
ω-3 fatty acids (%)	0.153	−0.374	0.613	−0.137	0.092	0.435	0.265	0.297	0.903	−0.033	0.663	0.118
ω-6/ω-3	0.206	0.334	0.321	0.223	0.983	0.006	0.634	−0.129	0.903	0.033	0.615	0.136
LA/ALA	0.085	0.444	0.274	0.291	0.585	0.148	0.453	0.202	0.424	0.215	0.250	0.305
EPA + DHA fatty acids (%)	0.160	−0.369	0.223	−0.323	0.464	−0.197	0.105	0.420	0.311	−0.271	0.586	0.147
Follicular fluid												
ω-6 fatty acids (%)	-	-	0.274	−0.482	0.635	0.129	-	-	0.263	0.491	0.324	−0.263
ω-3 fatty acids (%)	-	-	0.465	0.334	0.765	−0.081	-	-	0.170	−0.582	0.787	0.073
ω-6/ω-3	-	-	0.465	−0.334	0.853	−0.050	-	-	0.350	0.418	0.260	−0.299
LA/ALA	-	-	0.751	0.148	0.939	−0.021	-	-	0.816	0.109	0.531	−0.169
EPA + DHA fatty acids (%)	-	-	0.034	0.532	0.260	0.300	-	-	0.443	−0.207	0.958	−0.014

Spearman’s Rank Correlation Coefficient (ρ), * *p* < 0.05. MEDLIFE: The Mediterranean Lifestyle index; MDQI: Mediterranean Diet Quality Index; LA/ALA: Linoleic Acid/Alpha-Linolenic Acid; EPA + DHA: Eicosapentaenoic Acid + Docosahexaenoic Acid; LA: Linoleic Acid 18:2 (ω-6) (%); ALA: Alpha-Linolenic Acid 18:3 (ω-3) (%); EPA: Eicosapentaenoic Acid 20:5 (ω-3) (%); DHA: Docosahexaenoic Acid 22:6 (ω-3) (%).

**Table 6:** In Vitro Fertilization (IVF) Outcome Parameters.

IVF Parameters	Intervention Group (*n* = 7)		Control Group (*n* = 16)		
	$\bar{x}$ ± SS (Min–Max)	Median (IQR)	$\bar{x}$ ± SS (Min–Max)	Median (IQR)	*p*
Total Number of Oocytes	6.8 ± 4.51 (0.0–15.0)	5.0 (10.0)	7.76 ± 5.67 (0.0–20.0)	6.0 (8.5)	0.871
Number of MII Oocytes	6.4 ± 3.16 (0.0–13.0)	5.6 (5.0)	7.1 ± 5.36 (0.0–20.0)	5.0 (4.8)	0.769
Number of ICSI Procedures	3.6 ± 4.03 (0.0–13.0)	5.0 (5.0)	6.2 ± 5.37 (0.0–20.0)	5.0 (4.8)	0.974
Maturation Rate	88.4 ± 16.61 (0.0–100.0)	86.6 (40.0)	77.9 ± 34.58 (0.0–100.0)	81.0 (21.7)	0.624
Number of Pronuclear (PN) Oocytes	4.4 ± 3.60 (0.0–9.0)	5.0 (8.0)	4.81 ± 5.16 (0.0–17.0)	2.0 (5.8)	0.922
Fertilization Rate	65.0 ± 45.77 (0.0–100.0)	85.7 (100.0)	61.7 ± 35.8 (0.0–100.0)	63.3 (58.4)	0.671
Number of Embryos	2.14 ± 2.85 (0.0–6.0)	0.0 (6.0)	1.5 ± 2.1 (0.0–6.0)	0.0 (3.0)	0.769
Number of Embryo Transfers	0.3 ± 0.49 (0.0–1.0)	0.0 (0.1)	0.5 ± 0.6 (0.0–2.0)	0.0 (1.0)	0.535

Mann–Whitney U test, *p* < 0.05; IUI: Intrauterine Insemination; IVF/ICSI: In Vitro Fertilization/Intracytoplasmic Sperm Injection.

**Table 7:** Correlation with MEDLIFE and MDQI vs. IVF parameters.

	IVF Parameters		Total Number of Oocytes		Number of MII Oocytes		Number of Pronuclear (PN) Oocytes		Maturation Rate		Fertilization Rate	
			I	C	I	C	I	C	I	C	I	C
Dietary	MEDLIFE	r	0.311	−0.090	−0.198	−0.351	−0.343	−0.180	−0.364	0.135	−0.397	0.193
		*p*	0.241	0.741	0.609	0.239	0.366	0.538	0.336	0.644	0.289	0.508
	MDQI	r	0.477	−0.102	0.797	−0.084	0.741	0.026	−0.009	−0.311	−0.202	0.035
		*p*	0.164	0.718	0.010 *	0.785	0.022 *	0.929	0.981	0.279	0.603	0.905
	ω-6/ω-3	r	0.029	0.291	0.014	0.246	0.118	0.316	−0.050	0.031	−0.002	0.180
		*p*	0.914	0.275	0.959	0.359	0.663	0.234	0.854	0.910	0.995	0.505
	LA/ALA	r	0.057	0.317	0.029	0.216	0.104	0.245	−0.025	−0.114	0.008	0.068
		*p*	0.834	0.231	0.914	0.422	0.702	0.360	0.927	0.675	0.977	0.801
	EPA + DHA (%)	r	−0.015	0.025	−0.089	−0.319	−0.141	−0.027	−0.164	−0.277	0.002	0.205
		*p*	0.955	0.928	0.744	0.29	0.602	0.920	0.544	0.300	0.993	0.447
Serum	ω-6 (%)	r	0.439	0.000	0.239	−0.088	0.131	−0.103	0.000	−0.232	−0.052	−0.117
		*p*	0.116	1.000	0.372	0.745	0.629	0.704	1.000	0.387	0.847	0.665
	ω-3 (%)	r	0.487	−0.467	0.384	−0.637	0.410	−0.532	0.066	−0.201	0.264	−0.042
		*p*	0.078	0.079	0.142	0.008 *	0.114	0.034	0.809	0.456	0.323	0.878
	ω-6/ω-3	r	−0.072	0.131	−0.153	0.163	−0.324	0.159	0.008	−0.104	−0.335	−0.043
		*p*	0.806	0.642	0.571	0.546	0.220	0.557	0.977	0.702	0.205	0.874
	LA/ALA	r	0.606	−0.192	0.530	−0.229	0.700	−0.329	−0.669	−0.421	0.026	−0.143
		*p*	0.064	0.494	0.142	0.451	0.004 *	0.251	0.006 *	0.134	0.946	0.626
	EPA + DHA (%)	r	0.171	−0.475	0.240	−0.271	0.260	−0.148	0.251	0.027	0.263	0.311
		*p*	0.558	0.073	0.371	0.310	0.332	0.583	0.348	0.921	0.325	0.240
Follicular Fluid	ω-6 (%)	r	−0.414	0.213	−0.360	0.006	−0.198	0.126	−0.148	0.140	−0.243	0.082
		*p*	0.355	0.445	0.427	0.982	0.670	0.643	0.751	0.606	0.599	0.763
	ω-3 (%)	r	0.306	−0.587	0.252	−0.502	0.126	−0.177	0.000	0.075	0.430	0.267
		*p*	0.504	0.022 *	0.585	0.048	0.788	0.513	1.000	0.783	0.335	0.317
	ω-6/ω-3	r	−0.487	0.434	−0.414	0.453	−0.270	0.253	0.074	0.198	−0.318	−0.103
		*p*	0.268	0.106	0.355	0.078	0.558	0.345	0.875	0.462	0.487	0.705
	LA/ALA	r	−0.721	0.242	−0.348	0.234	−0.257	−0.097	0.751	−0.216	0.395	−0.372
		*p*	0.068	0.385	0.499	0.442	0.623	0.742	0.141	0.459	0.439	0.190
	EPA + DHA (%)	r	0.304	0.016	0.392	−0.102	0.383	−0.025	0.420	0.222	0.429	−0.123
		*p*	0.291	0.954	0.133	0.706	0.143	0.928	0.105	0.409	0.097	0.650

Spearman’s Rank Correlation Coefficient (ρ), * *p* < 0.05. C: Control group; I: Intervention group (last); MEDLIFE: The Mediterranean Lifestyle index; MDQI: Mediterranean Diet Quality Index.

**Table 8:** Correlation Between Dietary Intake Records and Serum Fatty Acid Levels.

			Dietary					Serum					Follicular Fluid				
			**ω-6 (%)**	**ω-3 (%)**	**ω-6/ω-3**	**LA/ALA**	**EPA + DHA %**	**ω-6 (%)**	**ω-3 (%)**	**ω-6/ω-3**	**LA/ALA**	**EPA + DHA %**	**ω-6 (%)**	**ω-3 (%)**	**ω-6/ω-3**	**LA/ALA**	**EPA + DHA %**
Dietary	ω-6 (%)	r						0.282	−0.192	−0.219	−0.175	−0.065	0.082	0.004	0.093	0.162	0.304
		*p*						0.192	0.379	0.315	0.425	0.767	0.71	0.986	0.673	0.46	0.159
	ω-3 (%)	r						−0.016	0.221	−0.242	0.061	−0.007	−0.373	0.177	−0.369	−0.393	−0.021
		*p*						0.943	0.311	0.266	0.781	0.976	0.08	0.419	0.084	0.063	0.925
	ω-6/ω-3	r						0.065	−0.36	0.218	−0.114	−0.071	0.315	−0.215	0.391	0.433	0.056
		*p*						0.767	0.092	0.317	0.606	0.746	0.143	0.325	0.065	0.039 *	0.8
	LA/ALA	r						−0.076	−0.39	0.25	−0.172	−0.032	0.326	−0.222	0.402	0.415	0.064
		*p*						0.732	0.066	0.25	0.433	0.886	0.129	0.309	0.057	0.049	0.772
	EPA + DHA (%)	r						0.005	−0.189	0.211	0.184	0.315	0.239	0.137	0.153	0.08	0.208
		*p*						0.982	0.399	0.347	0.414	0.143	0.284	0.543	0.497	0.723	0.341
Serum	ω-6 (%)	r	0.282	−0.016	0.065	−0.076	0.005						−0.565	−0.09	−0.155	0.173	−0.289
		*p*	0.192	0.943	0.767	0.732	0.982						0.015 *	0.689	0.492	0.44	0.181
	ω-3 (%)	r	−0.192	0.221	−0.36	−0.39	−0.189						0.334	0.561	0.054	−0.124	0.269
		*p*	0.379	0.311	0.092	0.066	0.399						0.129	0.015 *	0.813	0.583	0.214
	ω-6/ω-3	r	−0.219	−0.242	0.218	0.25	0.211						−0.348	−0.263	0.085	0.208	−0.281
		*p*	0.315	0.266	0.317	0.25	0.347						0.112	0.238	0.7	0.352	0.195
	LA/ALA	r	−0.175	0.061	−0.114	−0.172	0.184						0.046	−0.353	0.287	−0.024	−0.313
		*p*	0.425	0.781	0.606	0.433	0.414						0.835	0.099	0.184	0.917	0.145
	EPA + DHA (%)	r	−0.065	−0.007	−0.071	−0.032	0.315						−0.448	0.569	−0.235	−0.026	0.153
		*p*	0.767	0.976	0.746	0.886	0.143						0.032 *	0.015	0.28	0.906	0.485
Follicular Fluid	ω-6 (%)	r	0.082	−0.373	0.315	0.326	0.239	−0.565	0.334	−0.348	0.046	−0.448					
		*p*	0.71	0.08	0.143	0.129	0.284	0.015 *	0.129	0.112	0.835	0.032 *					
	ω-3 (%)	r	0.004	0.177	−0.215	−0.222	0.137	−0.09	0.561	−0.263	−0.353	0.569					
		*p*	0.986	0.419	0.325	0.309	0.543	0.689	0.015*	0.238	0.099	0.015					
	ω-6/ω-3	r	0.093	−0.369	0.391	0.402	0.153	−0.155	0.054	0.085	0.287	−0.235					
		*p*	0.673	0.084	0.065	0.057	0.497	0.492	0.813	0.7	0.184	0.28					
	LA/ALA	r	0.162	−0.393	0.433	0.415	0.08	0.173	−0.124	0.208	−0.024	−0.026					
		*p*	0.46	0.063	0.039 *	0.049	0.723	0.44	0.583	0.352	0.917	0.906					
	EPA + DHA (%)	r	0.304	−0.021	0.056	0.064	0.208	−0.289	0.269	−0.281	−0.313	0.153					
		*p*	0.159	0.925	0.8	0.772	0.341	0.181	0.214	0.195	0.145	0.485					

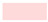
 No correlation; 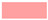 Weak negative correlation; 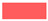 Moderate negative correlation; 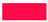 Strong negative correlation; 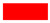 Very strong negative cor. 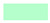 No correlation; 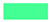 Weak positive correlation; 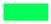 Moderate positive correlation; 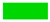 Strong positive correlation; 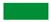 Very strong positive cor. Spearman’s Rank Correlation Coefficient (ρ), * *p* < 0.05; LA/ALA: Linoleic Acid/Alpha-Linolenic Acid; EPA + DHA: Eicosapentaenoic Acid + Docosahexaenoic Acid; LA: Linoleic Acid 18:2 (ω-6) (%); ALA: Alpha-Linolenic Acid 18:3 (ω-3) (%); EPA: Eicosapentaenoic Acid 20:5 (ω-3) (%); DHA: Docosahexaenoic Acid 22:6 (ω-3) (%).

## Data Availability

Available from the authors upon reasonable request.

## Supplementary Materials

The following supporting information can be downloaded at: https://www.mdpi.com/article/10.3390/medicina62030539/s1, Table S1. Daily energy requirements of the participants; Table S2. Energy and macronutrient content of a sample menu planned according to MEDLIFE portions and individual requirements; Table S3. Example menu prepared in accordance with Mediterranean diet principles; Table S4. Intrauterine insemination (IUI) and in vitro fertilization (IVF) protocols used in the study; Table S5. Sample Preparation Procedure; Table S6. Fatty acid groupings based on gas chromatography (GC) analysis; Table S7. Food group–based consumption amounts in the Mediterranean diet intervention pattern.

## Abbreviations

The following abbreviations are used in this manuscript:

**Abbreviation**	**Definition**
ACTH	Adrenocorticotropic hormone
AFC	Antral follicle count
ALA	Alpha-linolenic acid
AMH	Anti-Müllerian hormone
AMPK	5′-adenosine monophosphate-activated protein kinase
ARA	Arachidonic acid
ASRM	American Society for Reproductive Medicine
MEDLIFE-2014	Mediterranean Lifestyle Index
BMH	Basal metabolic rate
COC	Cumulus oophorus complex
CRH	Corticotropin-releasing hormone
PUFAs	Polyunsaturated fatty acids
DHA	Docosahexaenoic acid
WHO	World Health Organization
SFAs	Saturated fatty acids
EFORT	Exogenous FSH ovarian reserve test
EGF	Epidermal growth factor
EPA	Eicosapentaenoic acid
ESHRE	European Society of Human Reproduction and Embryology
FID	Flame ionization detector
FMF	Familial Mediterranean Fever
FSH	Follicle-stimulating hormone
FXR	Farnesoid X receptor
GAST	GnRH agonist stimulation test
GC	Gas chromatography
GH	Growth hormone
GIFT	Gamete intrafallopian transfer
GnRH	Gonadotropin-releasing hormone
Hcy	Homocysteine
HDL	High-density lipoprotein
HPG axis	Hypothalamic–pituitary–gonadal axis
HSG	Hysterosalpingography
ICMART	International Committee for Monitoring Assisted Reproductive Technologies
ICSI	Intracytoplasmic sperm injection
IGF	Insulin-like growth factor
IPAQ-SF	International Physical Activity Questionnaire–Short Form
IUI	Intrauterine insemination
IVF	In vitro fertilization
IVF-ET	In vitro fertilization–embryo transfer
LA	Linoleic acid
LDL	Low-density lipoprotein
LH	Luteinizing hormone
LXR	Liver X receptor
MDA	Malondialdehyde
MRI	Magnetic resonance imaging
mTORC1	Mammalian/mechanistic target of rapamycin complex 1
NPY	Neuropeptide Y
OA	Oleic acid
OAT	Oligoasthenozoospermia
OMI	Oocyte maturation inhibitor
OPU	Oocyte pick-up
PID	Pelvic inflammatory disease
PCOS	Polycystic Ovary Syndrome
PPAR	Peroxisome proliferator-activated receptor
TAG	Triacylglycerol
TEE	Total energy expenditure
TET	Tubal embryo transfer
TDHS	Turkey Demographic and Health Survey
TUBER	Turkish Dietary Guidelines
USG	Ultrasonography
