# Supplementary material for: Mediterranean Diet Adherence, Fatty Acid Profiles, and Early Assisted Reproduction Outcomes: Findings from a 12-Week Dietary Intervention
Source: Medicina (Kaunas). 2026 Mar 13;62(3):539. doi: 10.3390/medicina62030539 (PMC13028347; doi:10.3390/medicina62030539)
Supplement: Supplementary file 1 [file medicina-62-00539-s001.zip › medicina-4152949-supplementary.pdf]

Supplementary Materials

Table S1. Daily Energy Requirements of the Participants.

	Control group (n=16)		Intervention group (n=16)			
	x̄ ±SS (Min-Max)	Median (IQR)	x̄ ±SS (Min-Max)	Median (IQR)	**Z/t**	**p**
**BMR (kcal/day)**	1356,6±87,23 (1195,4-1535,8)	1357,8 (79,2)	1380,9±115,41 (1255,1-1713,4)	1346,4 (73,0)	0,170	0,865
**TEC (kcal/day)**	1628,0±104,62 (1434,5-1843,0)	1629,0 (95,1)	1657,1±138,5 (1506,1-2056,1)	1615,6 (87,6)	0,170	0,865
**Daily Energy Intake in the Mediterranean Diet**	1596,7±215,06* (1580,5-1810,0)	1600,0 (75,0)	1656,3±266,6 (1561,0-1922,3)	1600,0 (100,0)	-0,978	0,328

*** No dietary program was provided to the control group.**

Table S2. Energy and Macronutrient Content of a Sample Menu Planned According to MEDLIFE Portions and Individual Requirements.

		**1600 kcal/day**	**1700 kcal/day**	**1800 kcal/day**	**1900 kcal/day**
Energy (kcal)		1670	1750	1850	1950
Protein (%)		17	16	16	16
Carbohydrate (%)		45	48	48	46
Fat (%)		38	35	37	38
**Food groups**	**Portion Recommendations Based on the MEDLIFE**	**Portion**	**Portion**	**Portion**	**Portion**
Milk and Dairy Products (yy)	3 p/ day	3 p/day	3 p/ day	3 p/ day	3 p/ day
Red Meat and Poultry	2 p/week (2/7)	2 p/week	2 p/week	2 p/week	2 p/week
Fish	2 p/week (2/7)	2 p/week	2 p/week	2 p/week	2 p/week
Eggs	4 p/week (4/7)	4 p/week	4 p/week	4 p/week	4 p/week
Legumes	3 p/week (3/7)	3 p/week	3 p/week	3 p/week	3 p/week
Nuts and Seeds (including Olives)	1,5 p/ day	1,5 p/ day	1,5 p/day	1,5 p/day	1 p/day
Bread and Cereal Products	6-7 p/ day	5 p/ day	6 p/day	7 p/ day	7 p/ day
Vegetables	3 p/ day	2,5 p/ day	2,5 p/ day	3 p/ day	3 p/ day
Fruits	3-6 p/ day	3 p/ day	3 p/ day	3 p/ day	3 p/ day
Olive Oil	6-8 p/ day	6 p/ day	6 p/ day	7 p/ day	7 p/ day

Table S3. Example Menu in Accordance with the Mediterranean Diet Principles.

	1. menu	2.menu
Breakfast	____ servings of **milk** (semi-skimmed)<br>____ servings of **white cheese** (semi-skimmed)<br>____ **egg(s)**<br>**Salad** (dark green leafy vegetables, seasonal vegetables) *(with olive oil)*<br>____ **olives**<br>____ thin slices of **whole grain bread**	____ servings of **milk** (semi-skimmed)<br>____ servings of **white cheese** (semi-skimmed)<br>____ **egg(s)**<br>**Salad** (dark green leafy vegetables, seasonal vegetables) *(with olive oil)*<br>____ **olives**<br>____ thin slices of **whole grain bread**
Lunch	____ servings of **meat**<br>____ servings of **vegetable dish cooked with olive oil**<br>____ servings of **soup / rice / pasta**<br>____ servings of **yogurt** (semi-skimmed)<br>**Salad** (dark green leafy vegetables, seasonal vegetables) *(with olive oil)*<br>____ thin slices of **whole grain bread**	____ servings of **meat**<br>____ servings of **legume dish cooked with olive oil**<br>____ servings of **soup / rice / pasta**<br>____ servings of **yogurt** (semi-skimmed)<br>**Salad** (dark green leafy vegetables, seasonal vegetables) *(with olive oil)*<br>____ thin slices of **whole grain bread**
Snack	____ servings of **fruit**<br>____ servings of **milk group** (semi-skimmed)<br>____ servings of **nuts and seeds**<br>____ servings of **grains/cereal group**	____ servings of **fruit**<br>____ servings from the **milk group** (semi-skimmed)<br>____ servings of **nuts and seeds**<br>____ servings from the **cereal group**
Dinner	____ servings of **meat**<br>____ servings of **vegetable dish with olive oil**<br>____ servings of **soup / rice / pasta**<br>____ servings of **yogurt** (semi-skimmed)<br>**Salad** (dark green leafy vegetables, seasonal vegetables) *(with olive oil)*<br>____ thin slices of **whole grain bread**	____ servings of **meat**<br>____ servings of **vegetable dish cooked with olive oil**<br>____ servings of **soup / rice / pasta**<br>____ servings of **yogurt** (semi-skimmed)<br>**Salad** (dark green leafy vegetables, seasonal vegetables) *(with olive oil)*<br>____ thin slices of **whole grain bread**
Snack	____ servings of **fruit**<br>____ servings of **milk group** (semi-skimmed)<br>____ servings of **nuts and seeds**<br>____ servings of **grains/cereal group**	____ servings of **fruit**<br>____ servings from the **milk group** (semi-skimmed)<br>____ servings of **nuts and seeds**<br>____ servings from the **cereal group**

Daily Recommendations:

- **Olive Oil**: 30–40 mL/day (equivalent to 3–4 tablespoons)
- **Bread**: 120–160 g/day (4–6 thin slices, preferably whole grain)
- **Cereals and Grains**: 60 g/day (preferably whole grain varieties)
- **Vegetables**: 2 servings/day – one cooked and one raw (e.g., salad); 1 serving = 150–200 g
- **Milk**: 1 serving/day = 200 mL (low-fat or semi-skimmed)
- **Yogurt**: 1 serving/day = 150 mL (low-fat or semi-skimmed)
- **Olives**: 10 medium-sized olives/day
- **Nuts and Seeds**: 50 g/day (approximately one handful)
- **Water**: 6–8 glasses/day
- **Fruits**: 3–6 servings/day; 1 serving = 150–200 g fresh fruit or 100 mL (1 small glass) freshly squeezed fruit juice

Weekly Recommendations:

- **Cheese**: Preferably low-fat or semi-skimmed varieties
- **Red Meat**: Maximum once per week, up to 100–150 g
- **Large Fish**: Once per week, up to 100–150 g, or canned fish up to 50 g
- **Poultry**: Once per week, up to 100–150 g
- **Legumes**: At least twice per week; 1 serving = 8 tablespoons ≈ 150 g
- **Sweets**: Maximum 2 servings per week (e.g., 50 g sugar, 30 g chocolate, 40 g halva, or 4–6 biscuits)
- **Snacks**: Potato chips or popcorn, max 2 servings/week (1 pack or 50 g per serving)
- **Sugar-Sweetened Beverages**: Limit consumption

Lifestyle Recommendations:

- **Physical Activity**: At least 150 minutes per week or a minimum of 30 minutes per day
- **Sleep Duration**: 6–8 hours/day
- **Screen Time (TV)**: Less than 1 hour/day

Table S4. IUI and IVF Protocols

Intrauterine Insemination (IUI) Protocol

IUI was performed either during a natural cycle or following controlled ovarian hyperstimulation using fertility medications. When adequate follicular growth was achieved, ovulation was triggered with hCG administration. Subsequently, sperm was collected from the male partner, processed in the laboratory, and injected into the uterine cavity using a thin catheter under sterile conditions [25].

In Vitro Fertilization / Intracytoplasmic Sperm Injection (IVF/ICSI) Protocol

In women scheduled for IVF, controlled ovarian stimulation (COS) was initiated after the first clinical evaluation [25]. Initial and maintenance doses of gonadotropins were individualized based on patient age, BMI, baseline antral follicle count, FSH levels, and serial ultrasonographic assessments. COS protocols included recombinant FSH (Gonal-F®, Merck Serono, İstanbul, Türkiye), human menopausal gonadotropin (hMG) (Menogon®, 75 IU, Ferring, Türkiye; Menopur®, 75 IU, Ferring, Türkiye), or a combination of both. Ovarian stimulation started on the 2nd or 3rd day of the menstrual cycle. Follicular development typically continued for 10–12 days, with regular medication administration guided by ovarian response.

Oocyte Retrieval

The response was monitored via hormone levels (estradiol, LH, progesterone), serial transvaginal ultrasounds, and physical examinations. When at least two dominant follicles reached a diameter of 17–18 mm, ovulation was triggered with recombinant hCG (Ovitrelle®, 250 mcg syringe, Merck Serono, Türkiye). Approximately 34–38 hours later, follicular fluid and oocytes were aspirated transvaginally using a 16–17G needle under ultrasound guidance [26].

Sperm Collection

Semen samples were collected after 2–5 days of sexual abstinence [25].

Intracytoplasmic Sperm Injection (ICSI)

Oocytes were classified by embryologists as germinal vesicle, metaphase I, metaphase II (MII), or degenerated. All MII oocytes were fertilized using ICSI [28].

Embryo Transfer (ET)

On the 2nd or 3rd day after oocyte retrieval and ICSI, embryos were assessed for cleavage and quality based on blastomere count, morphology, and degree of fragmentation [27]. According to legal regulations, 1–2 embryos were transferred under ultrasound guidance either on Day 3 or Day 5. Luteal phase support was initiated following transfer. On Day 14 post-transfer, a pregnancy test was performed. If positive, luteal support was continued until the 12th week of gestation [3]. Quantitative serum β-hCG was measured on Day 12 post-transfer, and if >6 mIU/mL, the test was repeated after two days to confirm healthy progression of pregnancy.

Table S5. Sample Preparation Procedure

Sample Preparation for GC Analysis:

To precipitate proteins in serum and follicular fluid samples for gas chromatography (GC) analysis, 3 mL of cold acetone was added to 15 mL of sample and incubated at −20 °C for 15 minutes. The samples were then centrifuged at 4000 rpm for 5 minutes, and the supernatant was collected.

One milliliter of supernatant was refluxed at 90 °C with 5 mL of a MeOH:hexane:H_2SO_4 mixture (75:25:1, v/v/v). The resulting solution was transferred to a separating funnel and extracted with 10 mL of water and 10 mL of petroleum ether. After phase separation, the lower aqueous layer was discarded, and the upper organic layer was washed with 20 mL of water.

Following the removal of the aqueous phase, a spatula-tip amount of anhydrous sodium sulfate (Na_2SO_4) was added to the organic phase for drying. The solution was then filtered through filter paper and transferred into a test tube. The resulting fatty acid methyl esters (FAMEs) were stored at −20 °C until GC analysis.

Immediately before GC-FID analysis, the solvent was evaporated under a controlled nitrogen gas flow until 0.5 mL of sample remained. The residue was then dissolved in 0.25 mL of dichloromethane. The FAMEs in dichloromethane were transferred into glass inserts placed in GC vials, and 1 μL of the sample was injected into the GC-FID for analysis.

Table S5**. Fatty Acid Groupings Based on GC Analysis**

According to gas chromatography analysis, the total and ratio-based expressions of fatty acids in serum and follicular fluid were calculated as follows:

Total ω-6 Fatty Acids: Calculated as the sum of linoleic acid (18:2), gamma-linolenic acid (18:3), and arachidonic acid (20:4).

Total ω-3 Fatty Acids: Calculated as the sum of alpha-linolenic acid (18:3), eicosapentaenoic acid (EPA, 20:5), and docosahexaenoic acid (DHA, 22:6).

ω-6 / ω-3 Ratio: Determined by dividing the total ω-6 fatty acids (linoleic acid + gamma-linolenic acid + arachidonic acid) by the total ω-3 fatty acids (alpha-linolenic acid + EPA + DHA).

LA/ALA Ratio: Calculated as the ratio of linoleic acid (LA) to alpha-linolenic acid (ALA) based on measured concentrations.

EPA + DHA: Represents the combined total of two long-chain ω-3 fatty acids, which are precursors of eicosanoids, hormones that play important roles in various biological processes.

Table S6. Food Group-Based Consumption Amounts in the Mediterranean Diet Intervention Pattern.

	**Intervention group (first) (n=16)**		**Intervention group (second) (n=16)**		**Intervention group (third) (n=16)**		**Intervention group (last) (n=16)**		**Control group (n=16)**	
	$\bar{x}$±SS (Min-Max)	Median (IQR)	$\bar{x}$±SS (Min-Max)	Median (IQR)	$\bar{x}$±SS (Min-Max)	$\bar{x}$±SS (Min-Max)	Median (IQR)	$\bar{x}$±SS (Min-Max)	Median (IQR)	$\bar{x}$±SS (Min-Max)
**Milk and Dairy Products (g/day)**	150,8±77,72 (8,0-310,0)	135,0 (95,8)	153,7±93,58 (60,0-353,0)	133,3 (143,4)	168,4±98,15 (10,0-430,0)	140,0 (100,0)	135,7±68,68 (20,0-297,0)	136,7 (93,3)	162,3±91, (0,0-350,1)	156,7 (147,6)
	p¹=0,665 p²=0,659 p³=0,642 p⁴=0,429									
**Red Meat and Poultry (g/day)**	55,7±40,13 (0,0-136,7)	43,3 (53,4)	71,9±44,18 (0,0-132,7)	70,2 (72,5)	56,9±30,24 (0,0-118,8)	51,8 (43,4)	42,7±32,82 (0,0-110,0)	35,0 (45,9)	49,7±41,47 (0,0-130,4)	42,2 (85,8)
	p¹=0,835 p²=0,052 p³=0,663 p⁴=0,139									
**Fish (g/day)**	14,6±27,80 (0,0-83,3)	25,0 (0)	22,5±31,60 (0,0-80,0)	66,0 (0)	13,7±25,64 (0,0-70,0)	33,3 (0)	19,0±29,49 (0,0-70,0)	50,0 (0)	9,8±21,97 (0,0-66,7)	0,0 (0)
	p¹=0,074 p²=0,071 p³=0,496 p⁴=0,359									
**Eggs (g/day)**	40,2±16,21 (16,0-66,7)	36,7 (16,9)	34,5±19,11 (8,0-66,7)	33,3 (33,4)	30,3±16,90 (0,0-63,3)	33,3 (18,0)	39,0±16,25 (0,0-68,0)	33,3 (15,0)	33,1±19,75 (0,0-94,6)	27,5 (20,8)
	p¹=0,226 p²=0,345 p³=0,906 p⁴=0,125									
**Bread and Cereal Products (g/day)**	208,1±67,34 (125,5-348,0)	223,8 (117,8)	208,1±78,39 (93,3-403,0)	206,6 (109,7)	212,8±98,30 (58,5-366,0)	206,7 (172,5)	216,9±92,58 (50,0-388,0)	204,7 (90,9)	231,2±96,73 (63,8-428,0)	231,4 (102,3)
	p¹= 0,734 p²=0,584 p³=0,497 p⁴= 0,301									
**Legumes (g/day)**	33,2±24,02 (0,0-80,0)	30,0 (33,6)	23,8±22,99 (0,0-70,0)	21,0 (42,0)	24,0±17,86 (0,0-63,0)	20,0 (22,0)	26,2±20,90 (0,0-63,2)	32,0 (40,0)	8,8±8,28 (0,0-26,7)	10,0 (14,3)
	p¹=**0,001** p²=0,368 p³=0,408 p⁴=**0,019**									
**Nuts and Seeds (including Olives) (g/day)**	42,1±19,16 (10,0-70,0)	40,0 (34,2)	36,3±21,11 (8,0-93,3)	3,3 (16,0)	40,1±26,90 (3,3-102,0)	30,0 (34,4)	50,4±29,26 (15,0-118,0)	50,0 (47,0)	33,2±22,58 (0,0-94,5)	30,9 (30,1)
	p¹=0,141 p²=0,923 p³=0,201 p⁴=0,090									
**Fruits (g/day)**	185,5±63,24 (90,0-300,0)	181,0 (96,3)	143,3±116,42 (0,0-470,1)	140,0 (155,0)	167,2±108,94 (0,-373,30)	153,3 (196,6)	141,1±92,00 (0,0-306,7)	146,6 (150,0)	124,5±137,11 (0,0-429,1)	76,3 (207,4)
	p¹=**0,042** p²=0,068 p³=0,088 p⁴=0,336									
**Vegetables (g/day)**	170,6±96,91 (46,7-350,0)	154,6 (176,8)	217,4±85,95 (119,5-466,8)	208,3 (97,7)	229,1±99,86 (109,3-426,6)	223,3 (159,0)	262,3±117,27 (126,0-488,6)	260,0 (209,9)	216,1±166,44 (0,7-556,5)	185,9 (267,7)

	p^1=0,474 p^2=0,073 p^3=**0,004** p^4=0,274									
**Olive Oil (mL/day)**	12,1±7,53 (0,0-20,3)	9,8 (7,6)	16,8±10,77 (0,0-32,0)	20,00 (25,0)	23,50±9,63 (5,00-35,00)	30,0 (19,0)	20,8±9,35 (0,0-30,0)	25,0 (18,0)	9,5±8,51 (0,0-29,0)	7,5 (11,1)
	p^1=**0,006** p^2=0,553 p^3=0,906 p^4=**0,002**									

p^1: Baseline comparison between control and intervention groups, Mann–Whitney U test, $p<0,05$; p^2: Within-group comparison across all time points in the intervention group, Friedman, $p<0,05$; p^3: Pre- and post-intervention comparison within the intervention group, Wilcoxon, $p<0,05$; p^4: Post-intervention comparison between intervention and control groups, Mann–Whitney U test, $p<0,05$
